# Shoulder Arthroplasty Trials Are Infrequently Registered: A Systematic Review of Trials

**DOI:** 10.1371/journal.pone.0164984

**Published:** 2016-10-20

**Authors:** Matthew Thomas Sims, Zachary Carter Sanchez, James Murphy Herrington, James Barrett Hensel, Nolan Michael Henning, Caleb Josiah Scheckel, Matt Vassar

**Affiliations:** 1 Oklahoma State University Center for Health Sciences, Tulsa, Oklahoma, United States of America; 2 Oklahoma State University Medical Center, Tulsa, Oklahoma, United States of America; 3 Mayo Clinic, Phoenix, Arizona, United States of America; University of Sheffield, UNITED KINGDOM

## Abstract

**Introduction:**

With the intent of improving transparency in clinical research, the International Committee of Medical Journal Editors (ICMJE) established guidelines in 2005 regarding prospective clinical trial registration. This action worked to address bias related to selective outcome reporting in the medical literature. The objective of this study was to assess and characterize the quality of registration of clinical trials appearing in shoulder arthroplasty-related medical journals.

**Methods:**

All randomized trials involving human subjects, pertaining to shoulder arthroplasty, published between July 1, 2005 and December 31, 2015, and indexed in either PubMed or SportDISCUS were analyzed. We assessed the prevalence of registration, the timing of registration relative to patient enrollment periods, and the variable rates of orthopedic journal compliance with ICMJE and Food and Drug Administration clinical registration standards for our study.

**Results:**

Of the 382 articles identified, 345 (90.3%) were excluded due to failure to meet inclusion criteria. From the remaining 37, only 12 (32.4%) studies were found to be registered in a trial registry. Ten (10/12, 83.3%) of these provided their registration information within the body of the article. None of the included studies from ICMJE-recognized journals were registered. From 34 included studies from non-ICMJE recognized journals, 12 (35.3%) were registered.

**Conclusion:**

The level of compliance with clinical trial registration guidelines in the decade since their release among shoulder arthroplasty trials in orthopedic journals is poor. Given the importance of the issue, the prevalence of the problem, and the fact that many other medical specialties have already made efforts to improve ICMJE compliance, further work on the part of orthopedic surgery journal authors and editors is needed to ensure the publication of unbiased results.

**Trial Registration:**

UMIN000022487

## 1 Introduction

In an era where clinicians focus on evidence-based medicine to provide the highest level of patient care, clinicians increasingly rely upon well-constructed studies that yield accurate, relevant, and unbiased results. In medical research, evidence from randomized controlled trials are considered among the most reliable contribution to this foundation of knowledge upon which physicians rely to guide their clinical practices. Because of this, it is important that clinicians receive unbiased information as these studies have the potential to alter a physician’s practice and, therefore, have direct patient impact.

A clinical trial registry is a database that is publicly accessible and searchable by both the general public and scientific investigators alike and aids in the transparency of clinical trials. Information contained in registries include study parameters, details of an intervention, and the primary outcome(s) under investigation. The are several ways that trial registration promotes transparency [1]. It is known from prior research that studies with statistically significant results are more likely to be published than those lacking statistically significant findings. This action promotes redundancy in research and falsely elevates some interventions over others. By providing a public record of the actions of trialists, subsequent investigators have the means to evaluate trial registries for inconsistency between registered and published outcomes, alteration to periods of assessment, and retrospective outcome switching [2,3]—actions that are known to lead to publication bias [4,5]. For trial registries to effectively combat this bias, clinical trials must be registered and this registration must precede patient enrollment.

To further complicate matters, there have been instances of trialists measuring a multitude of variables and then capitalizing on chance occurrence for a statistically significant outcome. This practice is problematic since readers should be assured that the data reflects the true results from the experiment rather than a "data-mined" portrait of what the author wants to portray. Trial registration may help address this problem by refining the number of outcomes reported per trial to those most meaningful and important across investigations. This, in turn, could limit the number of outcomes being measured and minimize the occurrence of selective outcome [6] reporting bias or p-hacking [7]. If, for sake of illustration, a trial evaluated twenty variables, one is likely to be significant simply due to statistical probabilities. Pre-specification of outcomes in trial registries may limit this practice since there is a public record for accountability.

In 2005, the International Committee of Medical Journal Editors (ICMJE) stipulated that clinical investigators must register their clinical trials on a qualifying registry prior to patient enrollment as a pre-condition for publication among member journals [8–11]. In 2007, passage of the Food and Drug Administration (FDA) Amendments Act made trial registration for most drugs and devices in Phase 2–4 clinical trials the law [12]. These events resulted in a significant uptake in trial registration. For example, prior to the ICMJE mandate, Clinicaltrials.gov had only 13,153 registered trials. In contrast, ClinicalTrials.gov currently catalogues over 217,000 studies in 193 countries [13]. Trial registration, as delineated by the ICMJE, should include 20 items, including a description of the primary endpoints, assessment period, and funding sources [11,14,15]. Despite ICMJE requirements, completeness and quality of trial registry information remains an obstacle in a variety of subspecialties throughout medical literature [4,16]. This study extends the body of evidence to shoulder arthroplasty, a field in which trial registration compliance has yet to be explored.

## 2 Methods

We conducted a systematic review of randomized controlled trials to examine clinical trial registration reporting within high impact factor orthopedic journals. This study did not meet the regulatory definition of human subject research as defined in 45 CFR 46.102(d) and (f) of the Department of Health and Human Services’ Code of Federal Regulations and, therefore, was not subject to Institutional Review Board oversight. We applied relevant PRISMA guidelines (Checklist items 1–3, 5–11, 13, 17, 18, 24, 26, 27) for systematic reviews and SAMPL guidelines for reporting descriptive statistics to ensure best practices in reporting study information. This study was registered with the UMIN Clinical Trials Registry (UMIN000022487). Originally this study was designed to examine outcome reporting bias in shoulder arthroplasty publications; however, during data collection, there was a lack of registered trials and low response rate from corresponding authors. Therefore, the authors of this study amended the protocol of this study to examine the frequency of trial registration within shoulder arthroplasty publications. Data from this study is publicly available on figshare (https://dx.doi.org/10.6084/m9.figshare.3483116.v1).

### 2.1 Search Criteria

PubMed (S1 File) and SportDISCUS (S2 File) searches were conducted on June 3, 2016. The search was not limited to specific journals in order to thoroughly evaluate the current literature on shoulder arthroplasty interventions. Searches included the following PubMed MeSH Terms, “shoulder,” “shoulder pain,” “shoulder joint,” “athroplast*,” “hemiarthroplasty*,” “joint*,” “replace*,” “debridement,” “debrid*,” “surfac*,” “replac*,” and “resurfac*.” The search was limited to studies published between July 1, 2005 to December 31, 2015. The start date for the search was selected based on the ICMJE trial registration mandate stating that any clinical trial must be registered beginning July 1, 2005 [17].

### 2.2 Screening and Data Extraction

A library search specialist was used to generate search strategies and import the retrieved studies into an excel document. Three authors (MTS, ZCS, NMH) independently screened all of the articles within this excel document based on title and abstract. To qualify as a randomized controlled trial, a study had to include random assignment of participants into study conditions. Studies that met the following criteria were included for full-text review: RCTs, RCTs that used a crossover method, follow-up on previously performed RCTs that analyzed different primary outcomes published in the journals between July 1, 2005 and December 31, 2015. The exclusion criteria for this study was as follows: observational studies (including cohort, case-control, and cross sectional), systematic reviews/meta-analyses, ongoing studies, commentary or discussion pieces, articles with only a title or lacking an abstract, simulation-based studies, animal/ in vitro studies, cadaver studies, and studies not examining shoulder arthroplasty interventions. After screening was completed, a meeting was held to resolve differences by consensus. The authors designed an abstraction manual to ensure data coding was consistent and accurate. The abstraction manual was piloted based on a subset of randomized controlled trials, and a brief training session followed to reaffirm the intent of each coded element. Next the primary (MTS) and secondary (ZCS) authors assumed an equal number of randomized controlled trials. The following elements were abstracted: author name(s), article title, year of publication, journal title, whether or not the journal followed the ICMJE Recommendations [18], trial registration platform (ClinicalTrials.gov, WHO ICTRP, ISRCTN), trial registration identification number, time of trial registration (prospective, during patient recruitment, retrospective), method of acquiring trial registration number (within the article, via search of a trial registry platform, via email from the corresponding author), whether the corresponding author replied to email communication concerning registration, and discrepancies. In the case of a non-ICMJE recognized journal, the authors analyzed the specific journal’s “Instructions for Authors” to determine if the journal had a statement on trial registration for publication within their journal.

If no trial registration number was identified within an article, we searched ClinicalTrials.gov of the US National Institutes of Health, the World Health Organization International Clinical Trials Registry Platform (WHO ICTRP), and the International Standard Randomised Controlled Trial Number (ISRCTN) using the article title and authors’ names [19]. If a trial registration number was unable to be identified using the above search methods, we then contacted the corresponding authors to determine whether the randomized controlled trial had been registered and, if so, the name of the registry. In accordance with Dillman et al [20], we contacted each corresponding author twice in one week intervals in an attempt to obtain missing registration identification numbers. If this method failed to produce a trial registration number as well, we then considered the randomized controlled trial to be unregistered [21]. Any disagreements were settled by consensus.

### 2.3 Statistical Analysis

Descriptive statistics were used to summarize data. All analyses were conducted using STATA 13.1 (College Station, TX).

## 3 Results

**Fig 1:**
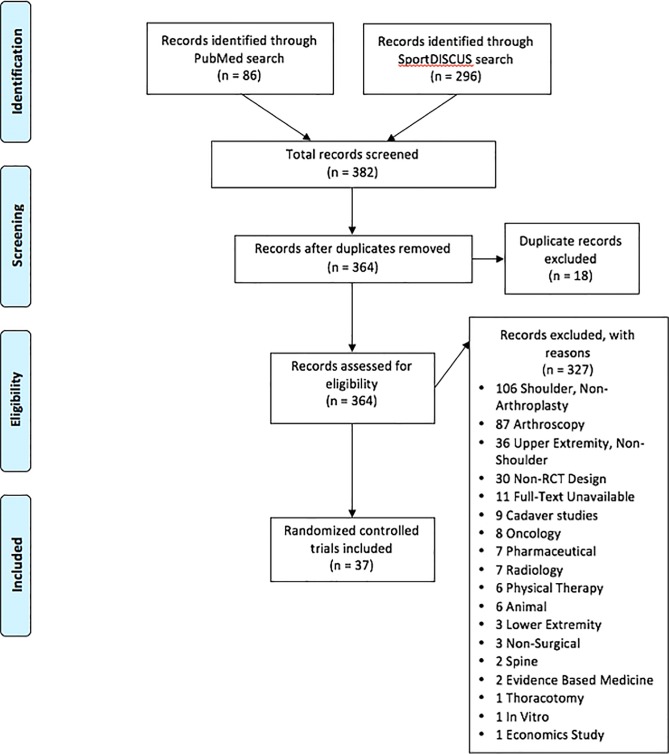
PRISMA Flowchart. PRISMA flowchart displaying the search results along with the included and excluded studies, with reasons.

We identified 382 RCTs published in PubMed and SportDISCUS published between January 1, 2005 and December 31, 2015 (Table 1). Of the 382 RCTs, 345 (90.3%) were excluded due to failure to meet inclusion criteria (Fig 1). Of the 37, three (3/37, 8.1%) were published within ICMJE recognized journals while the majority (34/37, 91.9%) were published in non-ICMJE recognized journals (Table 2). However, within the three ICMJE recognized publications, none of the articles provided clinical trial registration numbers or registered their trial, per our search (Table 3). Of the 34 RCTs published in non-ICMJE recognized journals, 12 (12/34, 35.3%) of the trials were registered. Ten (10/37, 27.0%) of the RCTs provided their trial registration identification number within the article, whereas two (2/37, 5.4%) were found via a search of registry platforms. Of the 12 (12/37, 32.4%) registered studies, 9 (9/12, 75.0%) were registered on ClinicalTrials.gov, 1 (1/12, 8.3%) was registered on ISRCTN, and 2 (2/12, 16.7%) were registered on the Netherlands Trial Registry. Furthermore, of the 12 registered studies, 5 (5/12, 41.7%) were prospectively registered, 4 (4/12, 33.3%) were retrospectively registered, 2 (2/12, 16.7%) were registered at the same time as patient recruitment began, and 1 (1/12, 8.3%) was unspecified as to when it was registered.

**Table 1:** Characteristics of included studies.

Characteristics	Number (%) of trials (n = 37)
*Date of Trial Publication*	
2005	1 (2.7)
2006	2 (5.4)
2007	4 (10.8)
2008	2 (5.4)
2009	3 (8.1)
2010	2 (5.4)
2011	3 (8.1)
2012	9 (24.3)
2013	2 (5.4)
2014	5 (13.5)
2015	4 (10.8)
*Journal*	
Arch Orthop Trauma Surg	2 (5.4)
BMC Musculoskelet Disord	4 (10.8)
Clin Orthop Relat Res	4 (10.8)
Injury	1 (2.7)
Int Orthop	1 (2.7)
J Shoulder Elbow Surg	17 (45.9)
JBJS	7 (18.9)
J Extra Corpor Technol	1 (2.7)
Orthopedics	1 (2.7)
Trials	1 (2.7)
*Type of Procedure*	
Hemiarthroplasty (HA)	12 (32.4)
Total Shoulder Arthroplasty (TSA)	12 (32.4)
Reverse Shoulder Arthroplasty (RSA)	4 (10.8)
Glenoid Resurfacing (GR)	2 (5.4)
Multiple Procedures	7 (18.9)

**Table 2:** ICMJE recognized journals.

**ICMJE Recognized Journals**		
Clinical Orthopaedics & Related Research	Orthopedics	
**Non-ICMJE Recognized Journals**		
Archives of Orthopaedic and Trauma Surgery **(✘)**	BMC Musculoskeletal Disorders **(✓)**	Injury **(✘)**
International Orthopaedics **(✘)**	Journal of Shoulder & Elbow Surgery **(✘)**	Journal of Bone & Joint Surgery **(✓)**
Journal of ExtraCorporeal Technology **(✘)**	Trials **(✓)**	

✘ **=** no statement on trial registration found in the journal’s “Instructions to Authors”

✓ **=** recommended/required trial registration based on the non-ICMJE recognized journal’s “Instructions for Authors”

**Table 3:** Percentage of reported trial registration numbers.

	ICMJE Journal	Non-ICMJE Journal	Total
**PubMed Sample**	**n = 3**	**n = 32**	**n = 35**
Trial registration number reported	0/3 (0.0%)	10/32 (31.3%)	10/35 (31.3%)
Trial Registered	0/3 (0.0%)	12/32 (37.5%)	12/35 (37.5%)
**SportDISCUS Sample**	**n = 0**	**n = 2**	**n = 2**
Trial registration number reported	0/0 (0.0%)	0/2 (0.0%)	0/2 (0.0%)
Trial Registered	0/0 (0.0%)	0/2 (0.0%)	0/2 (0.0%)
**Total**	**n = 3**	**n = 34**	**n = 37**

As described in the methods section, if the trial registration was not reported within the manuscript or located during a search of the three registry platforms, we electronically contacted the corresponding authors. Twenty-four authors were contacted and seven (RR = 29.2%) responded. Of the seven authors that responded, all reported their trial being unregistered. In the scenario that a trial registry number could not be found during our search and the corresponding author failed to respond, the trial was considered to be unregistered.

**Fig 2:**
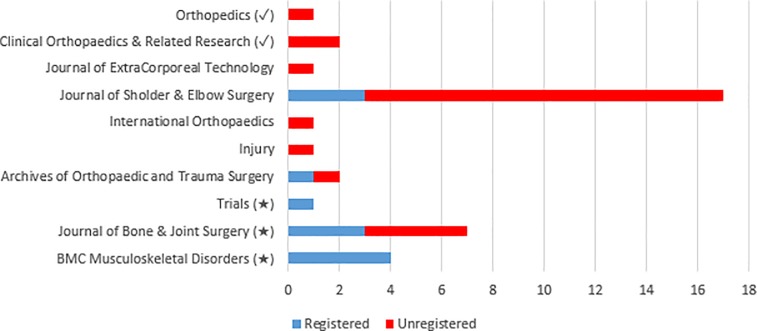
Trial registration frequency in ICMJE recognized and non-ICMJE recognized journals. (✓) = ICMJE recognized journal, (★) = non-ICMJE journal containing a trial registration statement within the journal’s “Instructions for Authors,” Journals lacking a symbol indicates that they are non-ICMJE recognized journals and that also lack a trial registration statement within the journal’s “Instructions for Authors”

The 34 RCTs published in non-ICMJE recognized journals occurred across 8 journals. Of the 8 journals, three (3/8, 37.5%) contained a statement within their “Instructions to Authors” referencing trial registration for publication within their journal (Table 3). Only one non-ICMJE recognized journal with their own trial registration statement, failed to follow their trial registration protocol within our sample (Fig 2). All of the ICMJE recognized journal publications were unregistered. Between July 1, 2005 and December 31, 2015, there were 25 (25/37, 67.6%) RCTs unregistered trials published: three (3/25, 12.0%) in ICMJE recognized journals, four (4/25, 16.0%) in non-ICMJE recognized journals with their own trial registration protocol statement, and 18 (18/25, 72.0%) in non-ICMJE recognized journals without a trial registration protocol statement.

Within this analysis we noted ten discrepancies in trial registration. In one case, a trial registration identification number (NCT00408096) was reported for two different studies with a different first author and title. Another discrepancy noted was a single trial being registered under two different registration numbers on different registry platforms. The trial reported its ClinicalTrial.gov trial identification number (NCT00158418) within the article, but was also found during a cursory search of the ISRCTN platform (ISRCTN75566721). Multiple studies had statuses other than “complete” on their clinical trial registration despite being published, such as, “active, not recruiting” (n = 2), “currently recruiting patients” (n = 2), “status: open—patient inclusion” (n = 1), “status: planned” (n = 1), and “unknown” (n = 2). Finally, one study listed its trial registration number within the manuscript despite the trial registration being withdrawn due to being “identified as being associated with a clinical device that has not been approved or cleared by the US Food and Drug Administration.”

## 4 Discussion

Of the 37 trials that met inclusion criteria for this study, 12 (35.3%) were registered. It is important to note that none of these 12 trials were from ICMJE member journals. Only three articles published in ICMJE journals met inclusion criteria, yet none were registered. The small sample of eligible ICMJE journal articles does limit the result; however, it is poignant to note that trials published in ICMJE member journals are not always registered despite the journals’ commitment to follow the trial registration policy.

Per the ICMJE’s *Recommendations for the Conduct*, *Reporting*, *Editing*, *and Publication of Scholarly Work in Medical Journals*, “the ICMJE requires, and recommends that all medical journal editors require, registration of clinical trials in a public trials registry at or before the time of first patient enrollment as a condition of consideration for publication [22]. Editors requesting inclusion of their journal on the ICMJE website list of publications that follow ICMJE guidance should recognize that the listing implies enforcement by the journal of ICMJE’s trial registration policy (p. 12).” Only 5 of the 12 registered trials in this study were prospectively registered. Four were retrospectively registered, 2 were registered during patient enrollment, and 1 was registered during an unspecified time. This raises two concerns. First, very few trials were registered or had registration numbers. Second, only a minority of trialists followed the recommendation of proper timing of registration. Registration after study completion allows trialists to make any modifications, including outcome switching, to the registry entry since the study may be completed at the time of registration. This practice could have significant consequences on clinical trial outcomes and clinical decisions based on such trials. Since a large portion of clinical trial registries still allow for retrospective registration, registration numbers at the time of manuscript submission is inadequate. Orthopedic journals must develop accountability mechanisms. Clinical trial registries must demarcate trials that were registered after patient enrollment [22]. The problem must be addressed by all stakeholders to enhance to integrity of orthopedic research and discourage unsound research practices.

While this study focuses primarily on the methodological practices of clinical trialists, it also calls attention to the completeness of reporting study information, which can have a significant impact on clinical practices. Current research literature has become a barrier to appropriate patient care in itself due to the lack of information provided within published reports [23]. The omission of this crucial information from the studies’ methodologies has prevented clinicians from being able to replicate these treatments. Following the publication of a recent chronic fatigue syndrome RCT, a trialist was frequently asked by clinicians for more in-depth information, necessitating the publication of a supplementary piece clarifying his graded exercise “prescription^”^ [23,24]. Furthermore, in an evaluation of 80 primary care and general medicine publications in *Evidence-Based Medicine*, greater than 50% of clinicians reported that elements of the interventions were missing in the published treatment description [23,25]. Thus, completeness and clarity of reporting—whether during trial registration or at publication—are foundational to good scientific practices, and actions should be taken to promote such practices throughout the research continuum.

In conclusion, trial registration is a requirement of ICMJE member journals and contributes to sound research practices. Trial registration also helps ensure that pre-specified trial outcomes are consistent with the published report and that outcome switching or selective reporting bias have not occurred. Stricter system of accountability for trial registration are needed to promote the integrity and validity of published research. Orthopedic journals and trial registries must take an active role in promoting and monitoring trial registration in order for research practices to improve.

## Supporting Information

S1 File PubMed Search String. (DOCX)

S2 File SportDISCUS Search String. (DOCX)

S3 File PRISMA Checklist. (DOC)

S1 Table Characteristics and full references of included studies. **TSA** = Total Shoulder Arthroplasty, **HA** = Hemiarthroplasty, **RSA** = Reverse Shoulder Arthroplasty, **GR** = Glenoid Resurfacing.
(DOCX)

S2 Table Excluded studies, with exclusion reasons. (DOCX)
