# Supplementary material for: Shoulder Arthroplasty Trials Are Infrequently Registered: A Systematic Review of Trials
Source: PLoS One. 2016 Oct 20;11(10):e0164984. doi: 10.1371/journal.pone.0164984 (PMC5072652; doi:10.1371/journal.pone.0164984)
Supplement: S1 File — (DOCX) [file pone.0164984.s001.docx]

**S1 File. PubMed Search String**

((((("shoulder"[MeSH Terms] OR "shoulder"[All Fields] OR "Shoulder Pain"[Mesh] OR "Shoulder Joint"[Mesh]))) AND (((((clinical[Title/Abstract] AND trial[Title/Abstract]) OR clinical trials as topic[MeSH Terms] OR clinical trial[Publication Type] OR random*[Title/Abstract] OR random allocation[MeSH Terms] OR therapeutic use[MeSH Subheading])) AND (arthroplast* OR hemiarthroplast* OR (joint* AND replace*) OR "Debridement"[Mesh] OR debrid* OR (surfac* AND replac*) OR resurfac*))) AND Randomized Controlled Trial[ptyp] AND ( "2005/07/01"[PDat] : "2015/12/31"[PDat] ) AND Humans[Mesh]))
