# Supplementary material for: Shoulder Arthroplasty Trials Are Infrequently Registered: A Systematic Review of Trials
Source: PLoS One. 2016 Oct 20;11(10):e0164984. doi: 10.1371/journal.pone.0164984 (PMC5072652; doi:10.1371/journal.pone.0164984)
Supplement: S2 File — (DOCX) [file pone.0164984.s002.docx]

**S2 File. SportDISCUS Search String**

| *#*  *Query*  *S8*  *S5 AND S6 AND S7*  *S7*  *TI (random* or factorial* or crossover* or cross-over* or placebo or “double blind” or “single blind” or assign* or allocat* or volunteer*) or AB (random* or factorial* or crossover* or cross-over* or placebo or “double blind” or “single blind” or assign* or allocat* or volunteer*) or SU (random* or factorial* or crossover* or cross-over* or placebo or “double blind” or “single blind” or assign* or allocat* or volunteer*) or KW (random* or factorial* or crossover* or cross-over* or placebo or “double blind” or “single blind” or assign* or allocat* or volunteer*)*  *S6*  *S1 OR S2 OR S3 OR S4*  *S5*  *TI shoulder* OR AB Shoulder* OR SU shoulder* OR KW shoulder**  *S4*  *KW arthro* OR KW surger* OR KW surgic* OR KW arthroplast* OR KW hemiarthroplast* OR KW debrid* OR KW resurfac**  *S3*  *SU arthro* OR SU surger* OR SU surgic* OR SU arthroplast* OR SU hemiarthroplast* OR SU debrid* OR SU resurfac**  *S2*  *AB arthro* OR AB surger* OR AB surgic* OR AB arthroplast* OR AB hemiarthroplast* OR AB debrid* OR AB resurfac**  *S1*  *TI arthro* OR TI surger* OR TI surgic* OR TI arthroplast* OR TI hemiarthroplast* OR TI debrid* OR TI resurfac** |
| --- |

*Limiters - Published Date: 20050701-20151231*
