# Supplementary material for: Shoulder Arthroplasty Trials Are Infrequently Registered: A Systematic Review of Trials
Source: PLoS One. 2016 Oct 20;11(10):e0164984. doi: 10.1371/journal.pone.0164984 (PMC5072652; doi:10.1371/journal.pone.0164984)
Supplement: S1 Table — TSA = Total Shoulder Arthroplasty, HA = Hemiarthroplasty, RSA = Reverse Shoulder Arthroplasty, GR = Glenoid Resurfacing. (DOCX) [file pone.0164984.s004.docx]

| **S1 Table. Characteristics and full references of included studies** | | | | |
| --- | --- | --- | --- | --- |
| **Study** | **Population** | **Gender** | **Intervention Types** | **Study Sample Size** |
| Agorstides et al 2007^1^ | Adult (>18) | Male and Female | HA | 49 |
| Alentorn-Geli et al 2014^2^ | Adult (>18) | Male and Female | HA, RSA | 32 |
| Barwood et al 2008^3^ | Unspecified | Unspecified | GR | 81 |
| Boons et al 2012^4^ | Adult (>18) | Male and Female | HA | 50 |
| Brorson et al^5^ | Adult (>18) | Unspecified | HA | Unspecified |
| Cai et al 2012^6^ | Adult (>18) | Male and Female | HA | 32 |
| Den Hartog et al 2010^7^ | Adult (>18) | Male and Female | HA | Unspecified |
| Ding et al 2015^8^ | Adult (>18) | Male and Female | TSA | 89 |
| Edwards et al 2010^9^ | Adult (>18) | Male and Female | TSA | 47 |
| Edwards et al 2007^10^ | Adult (>18) | Male and Female | TSA | 50 |
| Edwards et al 2012^11^ | Adult (>18) | Male and Female | RSA | 42 |
| Fialka et al 2008^12^ | Adult (>18) | Male and Female | HA | 40 |
| Fjalestad et al 2014^13^ | Adult (>18) | Unspecified | RSA | Unspecified |
| Greiner et al 2015^14^ | Adult (>18) | Male and Female | RSA | 34 |
| Gross et al 2011^15^ | Adult (>18) | Male and Female | TSA | 96 |
| Hendel et al 2012^16^ | Unspecified | Unspecified | TSA | 31 |
| Iannotti et al 2015^17^ | Unspecified | Unspecified | TSA | 46 |
| Kircher et al 2009^18^ | Unspecified | Unspecified | TSA | 20 |
| Lapner et al 2012^19^ | Adult (>18) | Male and Female | TSA, HA | 73 |
| Launonen et al 2012^20^ | Adult (>18) | Unspecified | HA | Unspecified |
| Litchfield et al 2011^21^ | Adult (>18) | Male and Female | TSA | 152 |
| Lo et al 2005^22^ | Adult (>18) | Male and Female | TSA, HA | 41 |
| Mechlenburg et al 2014^23^ | Adult (>18) | Male and Female | HA | 31 |
| Nuttall et al 2007^24^ | Adult (>18) | Male and Female | TSA | 20 |
| Olerud et al 2011^25^ | Adult (>18) | Male and Female | HA | 49 |
| Olerud et al 2012^26^ | Adult (>18) | Male and Female | HA | 55 |
| Poon et al 2014^27^ | Adult (>18) | Male and Female | RSA | 50 |
| Rahme et al 2006^28^ | Adult (>18) | Male and Female | TSA, HA | 24 |
| Rahme et al 2009^29^ | Adult (>18) | Male and Female | TSA | 27 |
| Rasmussen et al 2015^30^ | Adult (>18) | Male and Female | HA, GR | 40 |
| Sandow et al 2013^31^ | Adult (>18) | Male and Female | TSA, HA | 33 |
| Sebastia-Forcada et al 2014^32^ | Adult (>18) | Male and Female | HA, RSA | 62 |
| Soliman et al 2013^33^ | Adult (>18) | Male and Female | HA | 45 |
| Stilling et al 2012^34^ | Adult (>18) | Male and Female | GR | 21 |
| Tanaka et al 2006^35^ | Adult (>18) | Male and Female | TSA | 61 |
| Verbeek et al 2012^36^ | Adult (>18) | Unspecified | HA | Unspecified |
| Zavadil et al 2007^37^ | Adult (>18) | Male and Female | TSA | 38 |

**TSA** = Total Shoulder Arthroplasty, **HA** = Hemiarthroplasty, **RSA** = Reverse Shoulder Arthroplasty, **GR** = Glenoid Resurfacing

1. Agorastides I, Sinopidis C, El Meligy M, Yin Q, Brownson P, Frostick SP. Early versus late mobilization after hemiarthroplasty for proximal humeral fractures. *J Shoulder Elbow Surg;* 2007.
2. Alentorn-Geli E, Guirro P, Santana F, Torrens C. Treatment of fracture sequelae of the proximal humerus: comparison of hemiarthroplasty and reverse total shoulder arthroplasty. *Arch Orthop Trauma Surg*; 2014.
3. Barwood S, Setter KJ, Blaine TA, Bigliani LU. The incidence of early radiolucencies about a pegged glenoid component using cement pressurization*. J Shoulder Elbow Surg*; 2008.
4. Boons HW, Goosen JH, Van Grinsven S, Van Susante JL, Van Loon CJ. Hemiarthroplasty for humeral four-part fractures for patients 65 years and older a randomized controlled trial. *Clin Orthop Relat Res*; 2012.
5. Brorson S, Olsen BS, Frich LH, Jensen SL, Johannsen HV, Sorensen AK, Hrobjartsson A. Effect of osteosynthesis, primary hemiarthroplasty, and non-surgical management for displaced four-part fractures of the proximal humerus in elderly: A multi-centre, randomised clinical trial. *Trials*; 2009.
6. Cai M, Tao K, Yang C, Li S. Internal fixation versus shoulder hemiarthroplasty for displaced 4-part proximal humeral fractures in elderly patients. Orthopedics; 2012.
7. Den Hartog D, Tuinebreijer WE, Polinder S, Van Beeck EF, Breederveld RS, Bronkhorst MWGA, Eerenberg JP, Rhemrev S, Roerdink WH, Schraa G, Van Der Vis HM, Van Thiel TPH, Patka P, Nijs S, Schep NWL. Primary hemiarthroplasty versus conservative treatment for comminuted fractures of the proximal humerus in the elderly (ProCon): A Multicenter Randomized Controlled trial. *BMC Musculoskelet Disord;* 2010.
8. Ding DY, Mahure SA, Akuoko JA, Zuckerman JD, Kwon YW. Total shoulder arthroplasty using a subscapularis-sparing approach: A radiographic analysis. *J Shoulder Elbow* Surg; 2015.
9. Edwards TB, Labriola JE, Stanley RJ, O'Connor DP, Elkousy HA, Gartsman GM. Glenoid component insertion in total shoulder arthroplasty: Comparison of three techniques for drying the glenoid before cementation. *J Shoulder Elbow Surg;* 2010.
10. Edwards TB, Sabonghy EP, Elkousy H, Warnock KM, Hammerman SM, O'Connor DP, Gartsman GM. *J Shoulder Elbow Surg;* 2007.
11. Edwards TB, Trappey GJ, Riley C, O'Connor DP, Elkousy HA, Gartsman GM. Inferior tilt of the glenoid component does not decrease scapular notching in reverse shoulder arthroplasty: Results of a prospective randomized study. *J Shoulder Elbow Surg; 2012.*
12. Fialka C, Stampfl P, Arbes S, Reuter P, Oberleitner G, Vecsei V. Primary hemiarthroplasty in four-part fractures of the proximal humerus: Randomized trial of two different implant systems. *J Shoulder Elbow Surg*; 2008.
13. Fjalestad T, Iversen P, Hole MO, Smedsrud M, Madsen JE. Clinical investigation for displaced proximal humeral fractures in the elderly: a randomized study of two surgical treatments: reverse total prosthetic replacement versus angular stable plate Philos (The DELPHI-trial). *BMC Musculoskelet Disord;* 2014.
14. Greiner S, Schmidt C, Herrmann S, Pauly S, Perka C. Clinical performance of lateralized versus non-lateralized reverse shoulder arthroplasty: a prospective randomized study. *J Shoulder Elbow Surg;* 2015.
15. Gross RM, High R, Apker K, Haggstrom J, Fehringer JA, Stephan J. Vacuum assist glenoid fixation: does this technique lead to a more durable glenoid component? *J Shoulder Elbow Surg;* 2011.
16. Hendel MD, Bryan JA, Barsoum WK, Rodriguez EJ, Brems JJ, Evans PJ, Iannotti JP. Comparison of patient-specific instruments with standard surgical instruments in determining glenoid component position: a randomized prospective clinical trial. *JBJS;* 2012.
17. Iannotti JP, Weiner S, Rodriguez E, Subhas N, Patterson TE, Jun BJ, Ricchetti ET. Three-dimensional imaging and templating improve glenoid implant positioning. *JBJS;* 2015.
18. Kircher J, Wiedemann M, Magosch P, Lichtenberg S, Habermeyer P. Improved accuracy of glenoid positioning in total shoulder arthroplasty with intraoperative navigation: A prospective-randomized clinical study. *J Shoulder Elbow Surg*; 2009.
19. Lapner PLC, Sabri E, Rakhra K, Bell K, Athwal GS. Comparison of lesser tuberosity osteotomy to subscapularis peel in shoulder arthroplasty. *JBJS;* 2012.
20. Launonen AP, Lepola V, Flinkkila T, Strandberg N, Ojanpera J, Rissanen P, Malmivaara A, Mattila VM, Elo P, Viljakka T, Laitinen M. Conservative treatment, plate fixation, or prosthesis for proximal humeral fracture. A prospective randomized study. *BMC Musculoskelet Disord;* 2012.
21. Litchfield RB, McKee MD, Balyk R, Mandel S, Holtby R, Hollinshead R, Drosdowech D, Wambolt SE, Griffin SH, McCormack R. Cemented versus uncemented fixation of humeral components in total shoulder arthroplasty for osteoarthritis of the shoulder: a prospective, randomized, double-blind clinical trial-A JOINTs Canada Project. *J Shoulder Elbow Surg;* 2012.
22. Lo IKY, Litchfield RB, Griffin S, Faber K, Patterson SD, Kirkley A. Quality-of-life outcome following hemiarthroplasty or total shoulder arthroplasty in patients with osteoarthritis. *JBJS;* 2005.
23. Mechlenburg I, Klebe TM, Dossing KV, Amstrup A, Soballe K, Stilling M. Evaluation of periprosthetic bone mineral density and postoperative migration of humeral head resurfacing implants: two-year results of a randomized controlled clinical trial. *J Shoulder Elbow Surg;* 2014.
24. Nuttall D, Haines JF, Trail II. A study of the micromovement of pegged and keeled glenoid components compared using radiostereometric analysis. *J Shoulder Elbow Surg;* 2007.
25. Olerud P, Ahrengart L, Ponzer S, Saving J, Tidermark J. Hemiarthroplasty versus nonoperative treatment of displaced 4-part proximal humeral fractures in elderly patients: A randomized controlled trial. *J Shoulder Elbow Surg;* 2011.
26. Olerud P, Ahrengart L, Ponzer S, Saving J, Tidermark J. Hemiarthroplasty Improved health-related quality of life more than nonoperative treatment in older patients with four-part proximal humeral fractures. *JBJS;* 2012.
27. Poon PC, Chou J, Young SW, Astley T. A comparison of concentric and eccentric glenospheres in reverse shoulder arthroplasty: a randomized controlled trial. *JBJS;* 2014.
28. Rahme H, Mattsson P, Wikblad L, Larsson S. Cement and press-fit humeral stem fixation provides similar results in rheumatoid patients. *Clin Orthop Relat Res*; 2006.
29. Rahme H, Mattsson P, Wikblad L, Nowak J, Larsson S. Stability of cemented in-line pegged glenoid compared with keeled glenoid components in total shoulder arthroplasty. *JBJS;* 2009.
30. Rasmussen JV, Olsen BS, Sorensen AK, Hrobjartsson A, Brorson. Resurfacing hemiarthroplasty compared to stemmed hemiarthroplasty for glenohumeral osteoarthritis: a randomised clinical trial. *Int Orthop;* 2015.
31. Sandow MJ, David H, Bentall SJ. Hemiarthroplasty vs total shoulder replacement for rotator cuff intact osteoarthritis: how do they fare after a decade? *J Shoulder Elbow Surg;* 2013.
32. Sebastia-Forcada E, Cebrian-Gomez R, Lizaur-Utrilla A, Gil-Guillen V. Reverse shoulder arthroplasty versus hemiarthroplasty for acute proximal humeral fractures. A blinded, randomized, controlled, prospective study. *J Shoulder Elbow Surg;* 2014.
33. Soliman OA, Koptan WMT. Proximal humeral fractures treated with hemiarthroplasty: Does tenodesis of the long head of the biceps improve results? *Injury;* 2013.
34. Stilling M, Mechlenburg I, Amstrup A, Soballe K, Klebe T. Precision of novel radiological methods in relation to resurfacing humeral head implants: assessment by radiostereometric analysis, DXA, and geometrical analysis. *Arch Orthop Trauma Surg;* 2012.
35. Tanaka N, Sakahashi H, Hirose K, Ishima T, Ishii S. Augmented subscapularis muscle transposition for rotator cuff repair during shoulder arthroplasty in patients with rheumatoid arthritis. *J Shoulder Elbow Surg;* 2006.
36. Verbeek PA, van den Akker-Scheek I, Wendt KW, Diercks RL. Hemiarthroplasty versus angle-stable locking compression plate osteosynthesis in the treatment of three- and four-part fractures of the proximal humerus in the elderly: design of a randomized controlled trial. *BMC Musculoskelet Disord;* 2012.
37. Zavadil DP, Satterlee CC, Costigan JM, Holt DW, Shostrom VK. Autologous platelet gel and platelet-poor plasma reduce pain with total shoulder arthroplasty. *J Extra Corpor Tech*; 2007.
