# Supplementary material for: Shoulder Arthroplasty Trials Are Infrequently Registered: A Systematic Review of Trials
Source: PLoS One. 2016 Oct 20;11(10):e0164984. doi: 10.1371/journal.pone.0164984 (PMC5072652; doi:10.1371/journal.pone.0164984)
Supplement: S2 Table — (DOCX) [file pone.0164984.s005.docx]

|  | |
| --- | --- |
| **S2 Table. Excluded studies, with exclusion reasons** | |
| **Study Information** | **Reason For Exclusion** |
| Abbot AE, Li X, Busconi BD. Arthroscopic treatment of concomitant superior labral anterior posterior (SLAP) lesions and rotator cuff tears in patients over the age of 45 years. Am J Sports Med. 2009 Jul;37(7):1358-62. doi: 10.1177/0363546509331940. Epub 2009 Apr 13. PubMed PMID: 19364887. | Arthroscopic |
| Abbot AE, Li X, Busconi BD. Arthroscopic treatment of concomitant superior labral anterior posterior (SLAP) lesions and rotator cuff tears in patients over the age of 45 years. Am J Sports Med. 2009 Jul;37(7):1358-62. doi: 10.1177/0363546509331940. Epub 2009 Apr 13. PubMed PMID: 19364887. | Duplicate |
| Abdulla, Sean Y.; Southerst, Danielle; Côté, Pierre; Shearer, Heather M.; Sutton, Deborah; Randhawa, Kristi; Varatharajan, Sharanya; Wong, Jessica J. et al. Is exercise effective for the management of subacromial impingement syndrome and other soft tissue injuries of the shoulder? A systematic review by the Ontario Protocol for Traffic Injury Management (OPTIMa) Collaboration. 2015 Manual Therapy | Systematic Review |
| Abrams, G.D.; Gupta, A. K.; Hussey, K.E. Arthroscopic repair of full-thickness rotator cuff tears with and without acromioplasty: randomized prospective trial with 2-year follow-up. 2014. American Journal of Sports Medicine. | Arthroscopic |
| Ainsworth, Roberta; Lewis, Jeremy S., Exercise therapy for the conservative management of full thickness tears of the rotator cuff: a systematic review. British Journal of Sports Medicine Apr2007, Vol. 41 Issue 4, p200 | Systematic Review |
| Akbar, Michael; Balean, Gabriel; Brunner, Manuela; Seyler, Thorsten M.; Bruckner, Thomas; Munzinger, Judith; Grieser, Thomas; Gerner, Hans J.; Loew, Markus, Prevalence of Rotator Cuff Tear in Paraplegic Patients Compared with Controls. Journal of Bone & Joint Surgery, American Volume Jan2010, Vol. 92-A Issue 1, p23 | Rotator Cuff |
| Alemanno F, Ghisi D, Fanelli A, Faliva A, Pergolotti B, Bizzarri F, Fanelli G. Tramadol and 0.5% levobupivacaine for single-shot interscalene block: Effects on postoperative analgesia in patients undergoing shoulder arthroplasty. 2012. Minervia Anestesiol. | Anesthesia |
| Allison, Garry T. Abdominal muscle feedforward activation in patients with chronic low back pain is largely unaffected by 8 weeks of core stability training. 2012. Journal of Physiotherapy. | Lumbar Pain |
| Alqunaee, Marwan; Galvin, Rose; Fahey, Tom, Diagnostic Accuracy of Clinical Tests for Subacromial Impingement Syndrome: A Systematic Review and Meta-Analysis. Archives of Physical Medicine & Rehabilitation Feb2012, Vol. 93 Issue 2, p229 | Systematic Review |
| Andersen, Christine; Winding, Trine N; Vesterby, Martin S, Acta. Development of simulated arthroscopic skills. Orthopaedica Feb2011, Vol. 82 Issue 1, p90 | Arthroscopic |
| Arai, Ryuzo; Kobayashi, Masahiko; Harada, Hideto; Tsukiyama, Hiroyuki; Saji, Takahiko; Toda, Yoshinobu; Hagiwara, Yoshihiro; Miura, Takashi; Matsuda, Shuichi. Anatomical study for SLAP lesion repair. Knee Surgery, Sports Traumatology, Arthroscopy Feb2014, Vol. 22 Issue 2, p435 | Arthroscopic |
| Archetti Netto N, Tamaoki MJ, Lenza M, dos Santos JB, Matsumoto MH, Faloppa F, Belloti JC. Treatment of Bankart lesions in traumatic anterior instability of the shoulder: a randomized controled trial comparing arthroscopy and open techniques. 2012. Arhroscopy. | Arthroscopic |
| Arnoczky, Steven P.; Castricini, Roberto; Longo, Umile Giuseppe; De Benedetto, Massimo; Panfoli, Nicola; Pirani, Piergiorgio, Zini, Raul; Maffulli, Nicola; Denaro, Vincenzo. Platelet-rich plasma augmentation of rotator cuff repair: Letter. 2011. American Journal of Sports Medicine | Rotator Cuff |
| Arora, Rohit; Lutz, Martin; Deml, Christian; Krappinger, Dietmar; Haug, Luzian; Gabl, Markus. A Prospective Randomized Trial Comparing Nonoperative Treatment with Volar Locking Plate Fixation for Displaced and Unstable Distal Radial Fractures in Patients Sixty-five Years of Age and Older. Journal of Bone & Joint Surgery, American Volume 12/7/2011, Vol. 93-A Issue 23, p2146. | Radius |
| Atesok, Kivanc; Doral, M. Nedim; Whipple, Terry; Mann, Gideon-Mei-Dan, Omer; Atay, O. Ahmet; Beer, Yiftah; Lowe, Joseph; Soudry, Michael; Schemitsch, Emil H.. Arhroscopy-assisted fracture fixation. 2011. Knee Surgery, Sports Traumatology, Arthroscopy. | Arthroscopic |
| Atroshi, Isam; Brogren, Elisabeth; Larsson, Gert-Uno; Kloow, Jan; Hofer, Manfred; Berggren, Anne- Marie, Acta. Wrist-bridging versus non-bridging external fixation for displaced distal radius fractures: A randomized assessor-blind clinical trial of 38 patients followed for 1 year. Orthopaedica Jun2006, Vol. 77 Issue 3, p445. | Radius |
| Avery DM, Gibson BW, Carolan GF. Surgeon-rated visualization in shoulder arthroscopy: a randomized blinded controlled trial comparing irrigation fluid with and without epinephrine. 2015. Arthroscopy. | Arthroscopic |
| Baker, Nancy Al; Moehling, Krissy K.; Rubinstein, Elaine N.; Wollstein, Ronit; Gustafson, Normal P.; Baratz, Mark. The comparative effectiveness of combined sumbrical muscle splints and stretches on symptoms and funciton in carpal tunnel syndrome. 2012. Archives of Physical Medicine and Rehabilitation. | Wrist/Hand |
| Balke, Maurice; Liem, D.; Dedy, N.; Thorwesten, L.; Balke, Maryam; Poetzl, W.; Marquardt, B. The laser-pointer assisted angle reproduction test for evaluation of proprioceptive shoulder function in patients with instability. Archives of Orthopaedic & Trauma Surgery Aug2011, Vol. 131 Issue 8, p1077. | Shoulder, Not Arthroplasty |
| Baums, Mike H.; Spahn, G.; Steckel, H.; Fischer, A.; Schultz, W.; Klinger, H. -M. Comparative evaluation of the tendon-bone interface contact pressure in different single- versus double-row suture anchor repair techniques. Knee Surgery, Sports Traumatology, Arthroscopy Dec2009, Vol. 17 Issue 12, p1466. | Arthroscopic |
| Baums, M.; Buchhorn, G.; Spahn, G.; Poppendieck, B.; Schultz, W.; Klinger, H.-M. Biomechanical characteristics of single-row repair in comparison to double-row repair with consideration of the suture configuration and suture material. Knee Surgery, Sports Traumatology, Arthroscopy Nov2008, Vol. 16 Issue 11, p1052 | Arthroscopic |
| Baums, M.; Schminke, B.; Posmyk, A.; Miosge, N.; Klinger, H.-M.; Lakemeier, S. Effect of single- and double-row rotator cuff repair at the tendon-to- bone interface: preliminary results using an in vivo sheep model. Archives of Orthopaedic & Trauma Surgery Jan2015, Vol. 135 Issue 1, p111. | Rotator Cuff |
| Baums, Mike H.; Geyer, Michael; Büschken, Meike; Buchhorn, Gottfried H.; Spahn, Gunter; Klinger, Hans-Michael. Tendon–bone contact pressure and biomechanical evaluation of a modified suture-bridge technique for rotator cuff repair. Knee Surgery, Sports Traumatology, Arthroscopy Jul2010, Vol. 18 Issue 7, p992. | Rotator Cuff |
| Baums, M. H.; Buchhorn, G. H.; Gilbert, F.; Spahn, G.; Schultz, W.; Klinger, H.-M. Initial load-to-failure and failure analysis in single- and double-row repair techniques for rotator cuff repair. Archives of Orthopaedic & Trauma Surgery Sep2010, Vol. 130 Issue 9, p1193 | Rotator Cuff |
| Beitzel, Knut; Obopilwe, Elifho; Chowaniec, David; Nowak, Michael; Hanypsiak, Bryan; Guerra, James; Arciero, Robert; Mazzocca, Augustus. Biomechanical properties of repairs for dislocated AC joints using suture button systems with integrated tendon augmentation. Knee Surgery, Sports Traumatology, Arthroscopy Oct2012, Vol. 20 Issue 10, p1927 | AC Joint |
| Beitzel, Knut; Obopilwe, Elifho; Chowaniec, David M.; Niver, Genghis E.; Nowak, Michael D.; Hanypsiak, Bryan T.; Guerra, James J.; Arciero, Robert A.; Mazzocca, Augustus D. Biomechanical Comparison of Arthroscopic Repairs for Acromioclavicular Joint Instability: Suture Button Systems Without Biological Augmentation. American Journal of Sports Medicine Oct2011, Vol. 39 Issue 10, p2218. | Arthroscopic |
| Beitzel, Knut; Chowaniec, David M.; McCarthy, Mary Beth; Cote, Mark P.; Russell, Ryan P.; Obopilwe, Elifho; Imhoff, Andreas B.; Arciero, Robert A.; Mazzocca, Augustus D. Stability of Double-Row Rotator Cuff Repair Is Not Adversely Affected by Scaffold Interposition Between Tendon and Bone. American Journal of Sports Medicine May2012, Vol. 40 Issue 5, p1148. | Rotator Cuff |
| Bergeson, Adam G.; Tashjian, Robert Z.; Greis, Patrick E.; Crim, Julia; Stoddard, Gregory J.; Burks, Robert T. Effects of Platelet-Rich Fibrin Matrix on Repair Integrity of At-Risk Rotator Cuff Tears. American Journal of Sports Medicine Feb2012, Vol. 40 Issue 2, p286. | Rotator Cuff |
| Bierry G, Huang AJ, Chang CY, Torriani M, Bredella MA. MRI findings of treated bacterial septic arthritis. Skeletal Radiol. 2012 Dec;41(12):1509-16. doi: 10.1007/s00256-012-1397-2. Epub 2012 Mar 20. PubMed PMID: 22430565. | Infection |
| Bisson, Leslie J.; Manohar, Leslie M.; Wilkins, Ryan D.; Gurske-Deperio, Jennifer; Ehrensberger, Mark T. Influence of Suture Material on the Biomechanical Behavior of Suture-Tendon Specimens. American Journal of Sports Medicine May2008, Vol. 36 Issue 5, p907. | Shoulder, Not Arthroplasty |
| Björnsson Hallgren, Hanna C.; Holmgren, Theresa; Öberg, Birgitta; Johansson, Kajsa; Adolfsson, Lars E. A specific exercise strategy reduced the need for surgery in subacromial pain patients. British Journal of Sports Medicine Oct2014, Vol. 48 Issue 19, p1. | Physical Therapy |
| Board, Timothy N.; Srinivasan, Makram S. The effect of irrigation fluid temperature on core body temperature in arthroscopic shoulder surgery. Archives of Orthopaedic & Trauma Surgery May2008, Vol. 128 Issue 5, p531. | Arthroscopic |
| Bottoni CR, Smith EL, Berkowit MJ. Arthroscopic versus open shoulder stabilization for recurrent anterior instability a prospective randomized clinical trial. 2006. American Journal of Sports Medicine. | Arthroscopic |
| Boyer, P.; Bouthors, C.; Delcourt, T.; Stewart, O.; Hamida, F.; Mylle, G.; Massin, P. Arthroscopic double-row cuff repair with suture-bridging: a structural and functional comparison of two techniques. Knee Surgery, Sports Traumatology, Arthroscopy Feb2015, Vol. 23 Issue 2, p478. | Rotator Cuff |
| Brais, Godefroy; Ménard, Jérémie; Mutch, Jennifer; Laflamme, G-Yves; Petit, Yvan; Rouleau, Dominique M. Transosseous braided-tape and double-row fixations are better than tension band for avulsion-type greater tuberosity fractures. Injury Jun2015, Vol. 46 Issue 6, p1007 | Shoulder, Not Arthroplasty |
| Brianza, Stefano; Plecko, Michael; Gueorguiev, Boyko; Windolf, Markus; Schwieger, Karsten. Biomechanical evaulation of a new fixation technique for internal fixation of three-part proximal humerus fractures in a novel cadaveric model. 2010. Clinical Biomechanics. | Cadaver |
| Briem, D.; Ruecker, A.H.; Neumann, J.; Gebauer, M.; Kendoff, D.; Gehrke, T. et al. 3D fluoroscopic navigated reaming of the glenoid for total shoulder arthroplasty (TSA). 2011. Computer Aided Surgery. | Cadaver |
| Brorson, Stig; Frich, Lars H; Winther, Annika; Hróbjartsson, Asbjørn, Acta. Locking plate osteosynthesis in displaced 4-part fractures of the proximal humerus. Orthopaedica Aug2011, Vol. 82 Issue 4, p475. | Shoulder, Not Arthroplasty |
| Brorson, Stig; Rasmussen, Jeppe Vejlgaard; Frich, Lars Henrik; Olsen, Bo Sanderhoff; Hróbjartsson, Asbjørn. Benefits and harms of locking plate osteosynthesis in intraarticular (OTA Type C) fractures of the proximal humerus: A systematic review. Injury Jul2012, Vol. 43 Issue 7, p999. | Systematic Review |
| Brown MT, Murphy FT, Radin DM, Davignon I, Smith MD, West CR. Tanezumab. reduces osteoarthritic hip pain: results of a randomized, double-blind, placebo-controlled phase III trial. Arthritis Rheum. 2013 Jul;65(7):1795-803. doi: 10.1002/art.37950. PubMed PMID: 23553790. | Hip |
| Bryant, Dianne; Litchfield, Robert; Sandow, Michael; Gartsman, Gary M.; Guyatt, Gordon; Kirkley, Alexandra. A COMPARISON OF PAIN, STRENGTH, RANGE OF MOTION, AND FUNCTIONAL OUTCOMES AFTER HEMIARTHROPLASTY AND TOTAL SHOULDER ARTHROPLASTY IN PATIENTS WITH OSTEOARTHRITIS OF THE SHOULDER. Journal of Bone & Joint Surgery, American Volume Sep2005, Vol. 87-A Issue 9, p1947. | Systematic Review |
| Buess, Eduard; Waibl, Bernhard; Sieverding, Marc; Halbgewachs, Jörg. Posteroinferior shoulder instability: clinical outcome of arthroscopic stabilization in 32 shoulders and categorization based on labral mapping. Archives of Orthopaedic & Trauma Surgery May2015, Vol. 135 Issue 5, p673. | Arthroscopic |
| Burks, R.T.; Crime, J.; Brown, N.; et al. A prospective randomized clinical trial comparing arthroscopic single and double-row rotator cuff repair. 2009. American Journal of Sports Medicine. | Arthroscopic |
| Caldow, Jonathon; Richardson, Martin; Balakrishnan, Subash; Sobol, Tony; Lee, Peter; Ackland, David. A cruciate suture technique for rotator cuff repair. Knee Surgery, Sports Traumatology, Arthroscopy Feb2015, Vol. 23 Issue 2, p619. | Rotator Cuff |
| Camarinos, James; Marinko, Lee. Effectiveness of manual physical therapy for painful shoulder conditions: a systematic review. 2009. Journal of Manual & Manipulative Therapy. | Systematic Review |
| Carli, Angelo; Vadalà, Antonio; Zanzotto, Edoardo; Zampar, Guido; Vetrano, Mario; Iorio, Raffaele; Ferretti, Andrea. Reparable rotator cuff tears with concomitant long-head biceps lesions: tenotomy or tenotomy/tenodesis? Knee Surgery, Sports Traumatology, Arthroscopy Dec2012, Vol. 20 Issue 12, p2553 | Rotator Cuff |
| Carr, Andrew J.; Murphy, Richard; Dakin, Stephanie G.; Rombach, Ines; Wheway, Kim; Watkins, Bridget; Franklin, Sarah L. Platelet-Rich Plasma Injection With Arthroscopic Acromioplasty for Chronic Rotator Cuff Tendinopathy. American Journal of Sports Medicine Dec2015, Vol. 43 Issue 12, p2891. | Arthroscopic |
| Carr A, Cooper C, Murphy R, Watkins B, Wheway K, Rombach I, Beard D. PARot--assessing platelet-rich plasma plus arthroscopic subacromial decompression in the treatment of rotator cuff tendinopathy. 2013. Trials. | Rotator Cuff |
| Castagna, Alessandro; Borroni, Mario; Delle Rose, Giacomo; Markopoulos, Nikolaos; Conti, Marco; Vinci, Enzo; Garofalo, Raffaele. Effects of posterior-inferior capsular plications in range of motion in arthroscopic anterior bankart repair: a prospective randomized clinical study. Knee Surgery, Sports Traumatology, Arthroscopy Feb2009, Vol. 17 Issue 2, p188. | Arthroscopic |
| Castagna, Alessandro; Borroni, Mario; Garofalo, Raffaele; Rose, Giacomo; Cesari, Eugenio; Padua, Roberto; Conti, Marco; Gumina, Stefano. Deep partial rotator cuff tear: transtendon repair or tear completion and repair? A randomized clinical trial. Knee Surgery, Sports Traumatology, Arthroscopy Feb2015, Vol. 23 Issue 2, p460. | Rotator Cuff |
| Castoldi, Filippo; Rossi, Roberto; Lollino, Nicola; Renzulli, Federico; Berrino, Elena; Rossi, Paolo. Coracoid transfer in Bristow–Latarjet procedure: does it modify the biceps muscle? Knee Surgery, Sports Traumatology, Arthroscopy Jan2008, Vol. 16 Issue 1, p81. | Biceps |
| Castricini, R.; Longo, U.G.; De Benedetto, M.; Panfoli, N.; Pirani, P; Zini, R.; Maffulli, N. et al. Platelet-Rich fibrin matrix augmentation did not improve recovery and healing more than nonaugmented. 2011. Journal of Bone and Joint Surgery. | Rotator Cuff |
| Castricini, Roberto; Longo, Umile Giuseppe; De Benedetto, Massimo; Panfoli, Nicola; Pirani, Piergiorgio; Zini, Raul; Maffulli, Nicola; Denaro, Vincenzo. Platelet-Rich plasma augmentation for arthroscopic rotator cuff repair. 2011. American Journal of Sports Medicine. | Rotator Cuff |
| Ceponis, Peter J.M.; Chan, Denise; Boorman, Richard S.; Hutchison, Carol; Mohtadi, Nicholas G.H. A randomized pilot validation of educational measures in teaching shoulder arthroscopy to surgical residents. 2007: Vol. 50 Issue 5. p. 387-393. | Arthroscopic |
| Chang, K.; Hung, C.; Han, D; Chen, W; Wang, T.; Chien, K. Early Versus Delayed Passive Range of Motion Exercise for Arthroscopic Rotator Cuff Repair: A Meta-analysis of Randomize Controlled Trials. 2015. American Journal of Sports Medicine. | Arthroscopic |
| Charron, Kevin M.; Schepsis, Anthony A.; Voloshin, Ilya. Arthroscopic distal clavicle resection in athletes: a prospective comparison of the direct and indirect approach. 2007. American Journal of Sports Medicine. | Clavicle |
| Chen, L.; Xu, Z.; Peng, J.; Xing, F. Wang, H.; Ziang, Z. Effectiveness and safety of arthroscopic versus open Bankart repair for recurrent anterior shoulder. 2015. Archives of Orthopaedic and Trauma Surgery. | Arthroscopic |
| Cho, Chul-Hyun; Song, Kwang-Soon; Min, Byung-Woo; Jung, Gu-Hee; Lee, Young-Kuk; Shin, Hong- Kwan. Efficacy of interscalene block combined with multimodal pain control for postoperative analgesia after rotator cuff repair. Knee Surgery, Sports Traumatology, Arthroscopy Feb2015, Vol. 23 Issue 2, p542. | Rotator Cuff |
| Cho, Chul-Hyun; Song, Kwang-Soon; Min, Byung-Woo; Lee, Kyung-Jae; Ha, Eunyoung; Lee, Yong- Chul; Lee, Young-Kuk. Multimodal approach to postoperative pain control in patients undergoing rotator cuff repair. Knee Surgery, Sports Traumatology, Arthroscopy Oct2011, Vol. 19 Issue 10, p1744 | Rotator Cuff |
| Cho, Nam Su; Ha, Jeong Han; Rhee, Yong Girl. Patient-controlled Analgesia after arthorscopic rotator cuff repair subacromial catheter versus. 2007. American Journal of Sports Medicine. | Rotator Cuff |
| CHRISTIANSEN, DAVID HØYRUP; FROST, POUL; FALLA, DEBORAH; HAAHR, JENS PEDER; FRICH, LARS HENRIK; SVENDSEN, SUSANNE WULFF. Responsiveness and Minimal Clinically Important Change: A Comparison Between 2 Shoulder Outcome Measures. ournal of Orthopaedic & Sports Physical Therapy Aug2015, Vol. 45 Issue 8, p620. | Observational |
| Chung, Seok Won; Oh, Joo Han; Gong, Hyun Sik; Kim, Joon Yub; Kim, Sae Hoon. Factors Affecting Rotator Cuff Healing After Arthroscopic Repair: Osteoporosis as One of the Independent Risk Factors. American Journal of Sports Medicine Oct2011, Vol. 39 Issue 10, p2099 | Arthroscopic |
| Clement ND, Watts AC, Phillips C, McBirnie JM. Short-Term Outcome After Arthroscopic Bursectomy Debridement of Rotator Cuff Calcific Tendonopathy With and Without Subacromial Decompression: A Prospective Randomized Controlled Trial. Arthroscopy. 2015 Sep;31(9):1680-7. doi: 10.1016/j.arthro.2015.05.015. Epub 2015. Jul 15. PubMed PMID: 26188787. | Rotator Cuff |
| Coghlan, J.A.; Forbes, A.; McKenzie, D.; et al. Efficacy of subacromial ropivacaine infusion for rotator cuff surgery: a randomized trial. 2009. Journal of Bone and Joint Surgery. | Rotator Cuff |
| Coley, Brian; Jolles, Brigitte M.; Farron, Alain; Bourgeois, Aline; Nussbaumer, François; Pichonnaz, Claude; Aminian, Kamiar. Outcome evaluation in shoulder surgery using 3D kinematics sensors. Gait & Posture Apr2007, Vol. 25 Issue 4, p523. | Shoulder, Not Arthroplasty |
| Dahm, Diane L. Is Open Stabilization Superior to Arthroscopic Stabilization for the Treatment of Recurrent Traumatic Anterior Shoulder Instability?Journal of Bone & Joint Surgery, American Volume 3/5/2014, Vol. 96 Issue 5, pe41. | Arthroscopic |
| Dahm, Diane L. Controversy remains regarding the optimal technique for arthroscopic rotator cuff repair. 2012. Journal of Bone and Joint Surgery. | Rotator Cuff |
| Damkjaer, L.; Peterson, T.; Juul-Kristensen, B. e American Society of shoulder and elbow therapists' rehabilitation guideline better than standard care when applied to Bankart-operated patients? a controlled study. 2015 Clinical Rehabilitation. | Arthroscopic |
| Daniels JD, Sun S, Zafereo J et al. Preventing shoulder pain after cardiac rhythm management device implantation: a randomized, controlled study. 2011. Pacing and Clinical Electrophysiology. | Anesthesia/Cardiovascular |
| Dawson, Jill; Rogers, Katherine; Fitzpatrick, Ray; Carr, Andrew. The Oxford shoulder score revisited. Archives of Orthopaedic & Trauma Surgery Jan2009, Vol. 129 Issue 1, p119. | Evidence-Based Medicine |
| De Groef, A.; Van Kampen, M.; Dieltjens, E. et al. Effectiveness of postoperative physical therapy for upper-limb impairments after breast cancer. 2015. Archives of Physical Medicine and Rehabilitation. | Cancer |
| de Groot, Jurriaan H.; Angulo, Sonia M.; Meskers, Carel G.M.; der Heijden-Maessen, Hélène C.M. van; Arendzen, J.(Hans) H. Reduced elbow mobility affects the flexion or extension domain in activities of daily living. Clinical Biomechanics Aug2011, Vol. 26 Issue 7, p713. | Elbow |
| Dewing, Christopher B.; McCormick, Frank; Bell, S. Josh; Solomon, Daniel J.; Stanley, Mark; Rooney, Timothy B.; Provencher, Matthew T. An Analysis of Capsular Area in Patients With Anterior, Posterior, and Multidirectional Shoulder Instability. American Journal of Sports Medicine Mar2008, Vol. 36 Issue 3, p515. | Radiology |
| Diab MA, Fernandez GN, Elsorafy K. Time and cost savings in arthroscopic subacromial decompression: the use of bipolar versus monopolar radiofrequency. Int Orthop. 2009 Feb;33(1):175-9. doi: 10.1007/s00264-008-0541-z. Epub 2008 Apr 15. PubMed PMID: 18414860; PubMed Central PMCID: PMC2899216. | Arthroscopic |
| Diop, Amadou; Maurel, Nathalie; Chang, Vivian K.; Kany, Jean; Duranthon, Louis-Denis; Grimberg, Jean. Tendon fixation in arthroscopic latissimus dorsi transfer for irreparable posterosuperior cuff tears: An in vitro biomechanical comparison of interference screw and suture anchors. Clinical Biomechanics Nov2011, Vol. 26 Issue 9, p904. | Arthroscopic |
| Dolkart, Oleg; Chechik, Ofir; Zarfati, Yaron; Brosh, Tamar; Alhajajra, Fadi; Maman, Eran. A single dose of platelet-rich plasma improves the organization and strength of a surgically repaired rotator cuff tendon in rats.  Archives of Orthopaedic & Trauma Surgery Sep2014, Vol. 134 Issue 9, p1271. | Animal |
| Dougherty, Christopher P.; Howard, Timothy. Cost-effectiveness in Orthopedics: Providing Essential Information to Both Physicians and Health Care Policy Makers for Appropriate Allocation of Medical Resources. Sports Medicine & Arthroscopy Review Sep2013, Vol. 21 Issue 3, p166. | Econimical |
| Du Plessis, M.; Eksteen, E.; Jenneker, A.; Kriel, E. et al. The effectiveness of continuous passive motion on range of motion, pain and muscle strength following rotator cuff repair: a systematic review. 2011. Clinical Rehabilitation. | Systematic Review |
| Dunn, Warren R.; Schackman, Bruce R.; Walsh, Colin; Lyman, Stephen; Jones, Edward C.; Warren, Russell F.; Marx, Robert G. VARIATION IN ORTHOPAEDIC SURGEONS' PERCEPTIONS ABOUT THE INDICATIONS FOR ROTATOR CUFF SURGERY. Journal of Bone & Joint Surgery, American Volume Sep2005, Vol. 87-A Issue 9, p1978. | Rotator Cuff |
| Ebata, Shigeto; Sato, Hirokazu; Ohba, Tetsuro; Ando, Takashi; Haro, Hirotaka. Postoperative intervertebral stabilizing effect after cervical laminoplasty.  Journal of Back & Musculoskeletal Rehabilitation 2015, Vol. 28 Issue 2, p303. | Cancer |
| M. M. F., Oliveira; M. S. C., Gurgel; M. S., Miranda; M. A., Okubo; L. F. A., Feijó; G. A., Souza. Efficacy of shoulder exercises on locoregional complications in women undergoing radiotherapy for breast cancer: clinical trial. Brazilian Journal of Physical Therapy / Revista Brasileira de Fisioterapia mar/abr2009, Vol. 13 Issue 2, p136. | Cancer |
| Elmlund AO, Kartus J, Rostgard-Christensen L, Sernert N, Magnusson L, Ejerhed L. A 7-year prospective, randomized, clinical and radiographic study after arthroscopic Bankart reconstruction using 2 different types of absorbable tack. 2009. American Journal of Sports Medicine. | Arthroscopic |
| Elmlund AO, Kartus J, Rostgard-Christensen L, Sernert N, Magnusson L, Ejerhed L. A 7-year prospective, randomized, clinical and radiographic study after arthroscopic Bankart reconstruction using 2 different types of absorbable tack. 2009. American Journal of Sports Medicine. | Duplicate |
| Esposito J, Schemitsch EH, Saccone M, Sternheim A. External fixation versus open reduction with plate fixation for distal radius fractures: A meta-analysis of randomised controlled trials. 2013. Injury. | Meta-Analysis/Radius |
| Fares, K.M.; Mohamed, S.A.; Abd El-Rahman, A.M.; Mohamed, A.A.; Amin, A.T. Efficacy and safety of intraperitoneal dexmedetomidine with bupivacaine in laproscopic colorectal cancer surgery, a randomized trial. 2015. Pain Medicine. | Cancer |
| Farshad-Amacker, Nadja; Buck, Florian; Farshad, Mazda; Pfirrmann, Christian; Gerber, Christian. Partial supraspinatus tears are associated with tendon lengthening. Knee Surgery, Sports Traumatology, Arthroscopy Feb2015, Vol. 23 Issue 2, p408 | Rotator Cuff |
| Favejee, M M; Huisstede, B M A; Koes, B W. Frozen shoulder: the effectiveness of conservative and surgical interventions—systematic review. British Journal of Sports Medicine Jan2011, Vol. 45 Issue 1, p49. | Systematic Review |
| Frosch, Stephan; Buchhorn, Gottfried; Hoffmann, Anja; Balcarek, Peter; Schüttrumpf, Jan; August, Florian; Stürmer, Klaus; Walde, Hans; Walde. Novel single-loop and double-loop knot stitch in comparison with the modified Mason-Allen stitch for rotator cuff repair. Knee Surgery, Sports Traumatology, Arthroscopy May2015, Vol. 23 Issue 5, p1552. | Rotator Cuff |
| Franceschi, Francesco Maria; Ruzzini, Laura; Longo, Umile Giuseppe; Martina, Francesca maria; Zobel, Bruno Beomonte; Maffulli, Nicola; Denaro, Vincenzo. Equivalent Clinical Results of Arthroscopic Single-Row and Double- Row Suture Anchor Repair for Rotator Cuff Tears A Randomized Controlled Trial. American Journal of Sports Medicine Aug 2007: Vol. 35 Issue 8. p. 1254-1260. | Arthroscopic |
| Franceschi, Fracesco; Longo, Umile Giuseppe; Ruzzini, Laura; Rizzello, Giacomo; Maffulli, Nicola; Denaro, Vincenzo. No advantaes in repairing a Type II Superior Labrum Anterior and Posterior (SLAP) Lesion when associated with rotator cuff repair in patients over age 50. 2008. American Journal of Sports Medicine. | Rotator Cuff |
| Frank, Rachel M.; Mall, Nathan A.; Gupta, Deepti; Shewman, Elizabeth; Wang, Vincent M.; Romeo, Anthony A.; Cole, Brian J.; Bach, Bernard R.; Provencher, Matthew T.; Verma, Nikhil N. Inferior Suture Anchor Placement During Arthroscopic Bankart Repair: Influence of Portal Placement and Curved Drill Guide. American Journal of Sports Medicine May2014, Vol. 42 Issue 5, p1182. | Arthroscopic |
| Gebremariam, Lukas; Hay, Elaine M.; van der Sande, Renske; Rinkel, Willem D.; Koes, Bart W.; Huisstede, Bionka M. A. Subacromial impingement syndrome—effectiveness of physiotherapy and manual therapy. British Journal of Sports Medicine Aug2014, Vol. 48 Issue 16, p1202. | Rotator Cuff |
| Gebremariam, L.; Hay, E.M.; Koes, B.W.; Huisstede, B.M. Effectiveness of Surgical and Postsurgical Interventions for the Subacromial Impingement Syndrome: A Systemaic Review. 2011. Archives of Physical Medicine and Rehabilitation. | Systematic Review |
| Giles, Joshua W.; Puskas, Gabor J.; Welsh, Mark F.; Johnson, James A.; Athwal, George S. Suture Anchor Fixation of Bony Bankart Fractures: Comparison of Single-Point With Double-Point “Suture Bridge” Technique. American Journal of Sports Medicine Nov2013, Vol. 41 Issue 11, p2624. | Arthroscopic |
| Gillespie, Robert; Shishani, Yousef; Streit, Jonathan; Wanner, J.R; McCrum, Christopher; Syed, Tanvir; Haas, Adam; Gobezie, Reuben. The Safety of Controlled Hypotension for Shoulder Arthroscopy in the Beach-Chair Position. Journal of Bone & Joint Surgery, American Volume 7/18/2012, Vol. 94-A Issue 14, p1284. | Arthroscopic |
| Gillespie R, Shishani Y, Joseph S, Streit JJ, Gobezie R. Neer Award 2015: A randomized, prospective evaluation on the effectiveness of tranexamic acid in reducing blood loss after total shoulder arthroplasty. J Shoulder Elbow Surg. 2015 Nov;24(11):1679-84. doi: 10.1016/j.jse.2015.07.029. PubMed PMID: 26480877. | Pharmacology |
| Gmat, Daniel T.; Kenan, Samuel; Steiner, German C., Acta. Osteoarticular allograft of the proximal humerus--histopathological study 18 years after implantation. Orthopaedica Dec2005, Vol. 76 Issue 6, p934. | Shoulder, Not Arthroplasty |
| Godin, J.; Sekiya, J.K. Systematic Review of Arthroscopic versus open repair for recurrent anterior sholder dislocations. 2011. A Multidisciplinary Approach. | Systematic Review |
| Godin, Jonathan; Sekiya, Jon K. Systematic Review of rehabilitation of versus operative stabilization for the trreatment of first-time anterior shoulder dislocations. 2010. Sports Health: A Multidisciplinary Approach. | Systematic Review |
| Goebel, S.; Stehle, J.; Schwemmer, U.; et al. Interscalene brachial plexus block for open-shoulder surgery: a randomized, double-blind, placebo-controlled trial between single shot anesthesia and patient-controlled catheter system. 2015 American Journal of Sports Medicine. | Anesthesia |
| Gomoll, Andreas H.; Yanke, Adam B.; Kang, Richard W.; Chubinskaya, Susan; Williams, James M.; Bach, Bernard R.; Cole, Brian J. Long-Term Effects of Bupivacaine on Cartilage in a Rabbit Shoulder Model.  American Journal of Sports Medicine Jan2009, Vol. 37 Issue 1, p72. | Animal |
| Grafstein, E.; Stenstrom, R.; Christenson, J. et al. A prospective randomized controlled trial comparing circumfrential casting and splinting in displaced Colles fractures. 2010. Canadian Journal of Emergency Medicine. | Wrist/Hand |
| Greenfield, Russel H. No preventive Manual - Lymphedema related to breast cancer. 2011. Alternative Medicine Alert | Cancer |
| Greiner, S.; Ide, J.; Van Noort, A.; Mochizuki, Y.; Ochi, H.; Marraffino, S.; Sridharan, S.; Rudicel, S.; Itoi, E. Local rhBMP-12 on an absorbable collage sponge as an adjuvant therapy for rotator cuff repair a phase 1, randomized, standard or care control, multicenter study. 2015 American Journal of Sports Medicine | Rotator Cuff |
| Grewal, Ruby; Athwal, George S.; MacDermid, Joy C.; Faber, Kenneth J.; Drosdowech, Darren S.; El- Hawary, Ron; King, Graham J. W. Single Versus Double-Incision Technique for the Repair of Acute Distal Biceps Tendon Ruptures. Journal of Bone & Joint Surgery, American Volume 7/3/2012, Vol. 94-A Issue 13, p1166. | Biceps |
| Gulotta LV. Reverse shoulder arthroplasty provided better functional outcomes than hemiarthroplasty for acute proximal humeral fractures. 2015. Journal of Bone and Joint Surgery. | Commentary |
| Gumina, Stefano; Campagna, Vincenz; Ferrazza, Giancarlo; Giannicola, Giuseppe; Fratalocchi, Francesco; Milani, Alessandra; Postacchini, Franco. Use of Platelet-Leukocyte Membrane in Arthroscopic Repair of Large Rotator Cuff Tears.  ournal of Bone & Joint Surgery, American Volume 8/1/2012, Vol. 94-A Issue 15, p1345. | Arthroscopic |
| Haahr, J. P.; Østergaard, S.; Dalsgaard, J.; Norup, K.; Frost, P.; Lausen, S.; Holm, E. A.; Andersen, J. H. ARTHROSCOPIC DECOMPRESSION AND PHYSIOTHERAPY HAVE SIMILAR EFFECTIVENESS FOR SUBACROMIAL IMPINGEMENT. Journal of Bone & Joint Surgery, American Volume Nov2005, Vol. 87-A Issue 11, p2595. | Arthroscopic |
| Haering, Diane; Blache, Yoann; Raison, Maxime; Begon, Mickael. Mechanical risk of rotator cuff repair failure during passive movements: A simulation-based study. Clinical Biomechanics Dec2015, Vol. 30 Issue 10, p1181. | Rotator Cuff |
| Hageman, M.G.; Jayakumar, P.; King, J.D.; Guitton, T.G.; Doornbery, J.N.; Ring, D. The factors influencing the decision making of operative treatment for proximal humeral fractures. 2015. Journal of Elbow and Joint Surgery. | Observational |
| Hak, A.; Rajaratnam, K; Ayeni, O.R.; Moro, J.; Peterson, D.; Sprague, S.; Bhandari, M. A Double-Blinded Placebo Randomized Controlled Trial Evaluating Short-tern Efficacy of Platelet-Rich Plasma in Reducing Postoperative Pain After Arthroscopic Rotator Cuff Repair: A Pilot Study. 2015. Sports Health: A Multidisciplinary Approach. | Arthroscopic |
| Han, S.S.; Lee, Y.H.; Oh, J.H.; Aminzai, S.; Kim, S.H. Randomized, controlled trial of multimodal shoulder injection or intravenous patient-controlled analgesia after arthroscopic rotator cuff repair. 2013. Knee Surgery, Sports Traumatology, Arthroscopy. | Arthroscopic |
| Hase, Kimitaka; Kamisako, Michiyo; Fujiwara, Toshiyuki; Tsuji, Tetsuya; Liu, Meigen. The Effect of Zaltoprofen on Physiotherapy for Limited Shoulder Movement in Breast Cancer Patients: A Single-Blinded Before-After Trial.  Archives of Physical Medicine & Rehabilitation Dec 2006: Vol. 87 Issue 12. p. 1618-1622. | Cancer |
| Heckman, D.S.; Hoover, S.A., Weinhold, P.S., Spang, J.T., Creighton R.A. Repair of lesser tuberosity osteotomy for shoulder arthroplasty: biomechanical evaluation of the Backpack and Dual Row techniques. 2011. Journal of Shoulder and Elbow Surgery. | Cadaver |
| Hendel, Michael D.; Bryan, Jason A.; Barsoum, Wael K.; Rodriguez, Eric J.; Brems, John J.; Evans, Peter J.; Iannotti, Joseph P. Comparison of Patient-Specific Instruments with Standard Surgical Instruments in Determining Glenoid Component Position A Randomized Prospective Clinical Trial.  Journal of Bone & Joint Surgery, American Volume 12/5/2012, Vol. 94-A Issue 23, p2167. | Duplicate |
| Henkus HE, de Witte PB, Nelissen RG, Brand R, van Arkel ER. Bursectomy compared with acromioplasty in the management of subacromial impingement syndrome: a prospective randomised study. J Bone Joint Surg Br. 2009 Apr;91(4):504-10. doi: 10.1302/0301-620X.91B4.21442. PubMed PMID: 19336812. | Arthroscopic |
| Henninger HB, Barg A, Anderson AE, Bachus KN, Tashjian RZ, Burks RT. Effect of deltoid tension and humeral version in reverse total shoulder arthroplasty: a biomechanical study. 2012. Journal of Shoulder and Elbow Surgery. | Cadaver |
| Hepp, Pierre; Osterhoff, Georg; Engel, Thomas; Marquass, Bastian; Klink, Thomas; Josten, Christoph. Biomechanical Evaluation of Knotless Anatomical Double-Layer Double-Row Rotator Cuff Repair: A Comparative Ex Vivo Study. American Journal of Sports Medicine Jul2009, Vol. 37 Issue 7, p1363. | Rotator Cuff |
| Hiemstra, Laurie A.; Sasyniuk, Treny M.; Mohtadi, Nicholas G.H.; Gordon H. Shoulder strength after open versus arthroscopic stabilization. 2008. American Journal of Sports Medicine. | Arthroscopic |
| Hirschhorn, Andrew. Questioning the role of targeted respiratory physiotherapy over and above a standard clinical pathway in the postoperative management of patients following open thoracotomy. Journal of Physiotherapy (Elsevier) Dec2011, Vol. 57 Issue 4, p256. | Thoracotomy |
| Ho JC, Youderian A, Davidson IU, Bryan J, Iannotti JP. Accuracy and reliability of postoperative radiographic measurements of glenoid anatomy and relationships in patients with total shoulder arthroplasty. 2013. Journal of Shoulder and Elbow Surgery. | Observational |
| Hofmeister EP, Kim J, Shin AY. Comparison of 2 methods of immobilization of fifth metacarpal neck fractures: a prospective randomized study. J Hand Surg Am. 2008 Oct;33(8):1362-8. doi: 10.1016/j.jhsa.2008.04.010. PubMed PMID: 18929202. | Wrist/Hand |
| Holmgren, Theresa; Björnsson Hallgren, Hanna; Öberg, Birgitta; Adolfsson, Lars; Johansson, Kajsa. Effect of specific exercise strategy on need for surgery in patients with subacromial impingement syndrome: randomised controlled study. British Journal of Sports Medicine Oct2014, Vol. 48 Issue 19, p1456. | Rotator Cuff |
| Holmgren, Theresa; Hallgren, Hanna Björnsson; Öberg, Birgitta; Adolfsson, Lars; Johansson, Kajsa. Republished research: Effect of specific exercise strategy on need for surgery in patients with subacromial impingement syndrome: randomised controlled study. British Journal of Sports Medicine Sep2013, Vol. 47 Issue 14, p908. | Rotator Cuff |
| Holmgren T, Oberg B, Sjoberg I, Johansson K. Supervised strengthening exercises versus home-based movement exercises after arthroscopic acromioplasty: a randomized clinical trial. 2012. Journal of Rehabilitation Medicine. | Arthroscopic |
| Hove, Leiv M.; Krukhaug, Yngvar; Revheim, Kare; Helland, Per; Finsen, Vilh. Dynamic compared with static external fixation of unstable fractures of the distal part of the radius: a prospective, randomized multicenter study. 2010. Journal of Bone and Joint Surgery. | Radius |
| Hoyek N, Di Rienzo F, Collet C, Hoyek F. The therapeutic role of motor imagery on the functional rehabilitation of a stage II shoulder impingement syndrome. 2014. Disability and Rehabilitation. | Impingement Syndrome |
| Huisstede, B. M. A.; Tashjian, Robert Z. [Commentary on] The Effectiveness of Nonoperative Treatment for Frozen Shoulder: A Systematic Review. Clinical Journal of Sport Medicine Mar2012, Vol. 22 Issue 2, p168. | Commentary |
| Huisstede, Bionka M.A.; Gebremariam, Lukas; van der Sande, Renske; Hay, Elaine M.; Koes, Bart W. Effectiveness of Interventions of Specific Complaints of the Arm, Neck, and/or Shoulder: 3 Musculoskeletal Disorders of the Hand. An Update. Archives of Physical Medicine & Rehabilitation Feb2010, Vol. 91 Issue 2, p298. | Systematic Review |
| Huisstede BM, Hoogvliet P, Paulis WD, van Middelkoop M. Effectiveness of interventions for secondary Raynaud's Phenomenon: A systematic review. 2011. Archives of Physical Medicine and Rehabilitation. | Raynaud's |
| Huisstede BM, van Middelkoop M, Randsdorp MS. Effectiveness of interventions of specific complaints of the arm, neck, and/or shoulder: 3 musculoskeletal disorders of the hand. An update. 2010. Archives of Physical Medicine and Rehabilitation. | Hand |
| Huisstede, B.M.A.; Koes, B.W.; Gabremariam, L.; et al. Current evidence for effectiveness of interventions to treat rotator cuff tears. 2011. American Journal of Sports Medicine. | Rotator Cuff |
| Hultenheim Klintberg I, Gunnarsson AC, Styf J, Karlsson J. Early activation or a more protective regime after arthroscopic subacromial decompression--a description of clinical changes with two different physiotherapy treatment protocols--a prospective, randomized pilot study with a two-year follow-up. 2008. Clinical Rehabilitation. | Duplicate |
| Mohtadi, N.G.; Hollinshead, R.M.; Sasyniuk, T.M.; fletcher, J.A.; Chan, D.S.; Li, F.X. A Randomized Clinical Trail Comparing Mini-open With Open Rotator Cuff Repair: Two-year Outcomes. (Abstract). Clinical Journal of Sport Medicine Mar 2006: Vol. 16 Issue 2. p. 182. | Rotator Cuff |
| Iannotti JP, Codsi MJ, Kwon YW, Derwin K, Ciccone J, Brems JJ. Porcine small intestine submucosa augmentation of surgical repair of chronic two-tendon rotator cuff tears. A randomized, controlled trial. 2006. Journal of Bone and Joint Surgery American. | Rotator Cuff |
| Ibrahim M, Donatelli R, Hellman M, Echternach J. Efficacy of a static progressive stretch device as an adjunct to physical therapy in treating adhesive capsulitis of the shoulder: a prospective, randomised study. 2014. Physiotherapy. | Adhesive Capsulitis |
| Ilfeld BM, Vandenborne K, Duncan PW, Sessler DI, Enneking FK, Shuster JJ et al. Ambulatory continuous interscalene nerve blocks decrease the time to discharge readiness after total shoulder arthroplasty: a randomized, triple-masked, placebo-controlled study. 2006. Anesthesiology | Anesthesia |
| Jacquot A, Dezaly C, Goetzmann T, Roche O, Sirveaux F, Mole D. Is rotator cuff repair appropriate in patients older than 60 years of age? prospective, randomised trial in 103 patients with a mean four-year follow-up. 2014 Orthopedic Trauma and Srugical Resuscitation. | Rotator Cuff |
| Jaehwa Kim; Juhwan Chung; Hyunsoo Ok. Asymptomatic acromioclavicular joint arthritis in arthroscopic rotator cuff tendon repair: a prospective randomized comparison study. Archives of Orthopaedic & Trauma Surgery Mar2011, Vol. 131 Issue 3, p363. | Arthroscopic |
| Jang, K.U.; Choi, J.S.; Mun, J.H.; Jeon, J.H.; Seo, C.H.; Kim, J.H. Multi-axis shoulder abduction splint in acute burn rehabillitation: a randomized controlled pilot trial. 2015 Clinical Rehabilitation. | Burn |
| Jo, Chris Hyunchul; Kim, Ji Eun; Yoon, Kang Sup; Lee, Ji Ho; Kang, Seung Baik; Lee, Jae Hyup; Han, Hyuk Soo; Rhee, Seung Hwan; Shin, Sue. Does Platelet-Rich Plasma Accelerate Recovery After Rotator Cuff Repair? A Prospective Cohort Study. American Journal of Sports Medicine Oct2011, Vol. 39 Issue 10, p2082. | Rotator Cuff |
| Jo CH, Shin JS, Lee YG, Shin WH, Kim H, Lee SY. Platelet-rich plasma for arthroscopic repair of large to massive rotator cuff tears: a randomized, single-blind, parallel-group trial. 2013. American Journal of Sports Medicine. | Rotator Cuff |
| Jo CH, Shin JS, Lee YG, Shin WH, Kim H, Lee SY. Platelet-rich plasma for arthroscopic repair of large to massive rotator cuff tears: a randomized, single-blind, parallel-group trial. 2013. American Journal of Sports Medicine. | Repeat |
| Jo, C.H.; Shin, J.S.; Shin, W.H.; et al. Platelet-rich plasma for arthroscopic repair of medium to large rotator cuff tears. 2015 American Journal of Sports Medicine. | Arthroscopic |
| Jorheim M, Isaxon I, Flondell M, Kalen P, Atroshi I. Short-term outcomes of trapeziometacarpal artelon implant compared with tendon suspension interposition arthroplasty for osteoarthritis: a matched cohort study. 2009. Journal of Hand Surgery, American. | Wrist/Hand |
| Karantana, Alexia; Downing, Nicholas D.; Forward, Daren P.; Hatton, Mark; Taylor, Andrew M.; Scammell, Brigitte E.; Moran, Chris G.; Davis, Tim R. C. Surgical treatment of distal radial fractures with a volar locking plate versus conventional percutaneous methods. 2013. Journal of Bone and Joint Surgery. | Radius |
| Kedgley AE, DeLude JA, Drosdowech DS, Johnson JA, Bicknell RT. Humeral head translation during glenohumeral abduction following computer-assisted shoulder hemiarthroplasty. 2008. Journal of Bone and Joint Surgery, British | Cadaver |
| Keener, Jay D.; Galatz, Leesa M.; Stobbs-Cucchi, Georgia; Patton, Rebecca; Yamaguchi, Ken. Rehabilitation Following Arthroscopic Rotator Cuff Repair: A Prospective Randomized Trial of Immobilization Compared with Early Motion.  Journal of Bone & Joint Surgery, American Volume 1/1/2014, Vol. 96 Issue 1, p11. | Arthroscopic |
| Ketola S, Lehtinen J, Rousi T, Nissinen M, Huhtala H, Arnala I. Which patients do not recover from shoulder impingement syndrome, either with operative treatment or with nonoperative treatment? Acta Orthop. 2015;86(6):641-6. doi:10.3109/17453674.2015.1033309. Epub 2015 Sep 24. PubMed PMID: 25809315; PubMed Central PMCID: PMC4750760. | Arthroscopic |
| Ketola, Saara; Lehtinen, Janne; Rousi, Timo; Nissinen, Maunu; Huhtala, Heini; Arnala, Ilkka, Acta. Which patients do not recover from shoulder impingement syndrome, either with operative treatment or with nonoperative treatment?  Orthopaedica Dec2015, Vol. 86 Issue 6, p641. | Rotator Cuff |
| Kim, Jae; Lee, Jae; Park, Chi. Extracorporeal shock wave therapy is not useful after arthroscopic rotator cuff repair. Knee Surgery, Sports Traumatology, Arthroscopy Dec2012, Vol. 20 Issue 12, p2567. | Arthroscopic |
| Kim, Kyung; Rhee, Yong; Park, Jin; Shin, Hyun; Cha, Soo; Park, Jun; Han, Sun; Yang, Jae. Anteroposterior translation of the glenohumeral joint in various pathologies: differences between shoulder MRI in the adducted neutral rotation and abducted externally rotated positions.  Knee Surgery, Sports Traumatology, Arthroscopy Sep2015, Vol. 23 Issue 9, p2611. | Radiology |
| Jae Kwang Kim; Young Do Koh; Seung Hwan Kook. Effect of Calcium Phosphate Bone Cement Augmentation on Volar Plate Fixation of Unstable Distal Radial Fractures in the Elderly. Journal of Bone & Joint Surgery, American Volume 4/6/2011, Vol. 93-A Issue 7, p609. | Radius |
| Kim, Kyung; Rhee, Yong; Park, Jin; Shin, Hyun; Cha, Soo; Park, Jun; Han, Sun; Yang, Jae. Anteroposterior translation of the glenohumeral joint in various pathologies: differences between shoulder MRI in the adducted neutral rotation and abducted externally rotated positions.  Knee Surgery, Sports Traumatology, Arthroscopy Sep2015, Vol. 23 Issue 9, p2611. | Duplicate |
| Kim, Y.S.; Chung, S.W.; Kim, J.Y.; et al. Is Early passive motion exercise necessary after arthroscopic rotator cuff repair? 2012. American Journal of Sports Medicine. | Rotator Cuff |
| Kim, Y.S.; Leen, H.J.; Park, I.J. Clinical outcomes do not support arthroscopic posterior capsular release in addition to anterior release for shoulder stiffness: a randomized controlled study. 2014. American Journal of Sports Medicine. | Arthroscopic |
| Klinger, Hans-Michael; Buchhorn, Gottfried H.; Heidrich, Gabert; Kahl, Enrico; Baums, Mike H. Biomechanical evaluation of rotator cuff repairs in a sheep model: Suture anchors using arthroscopic Mason-Allen stitches compared with transosseous sutures using traditional modified Mason-Allen stitches . Clinical Biomechanics Mar2008, Vol. 23 Issue 3, p291. | Arthroscopic |
| Klinger, Hans-Michael; Steckel, Hanno; Spahn, Gunter; Buchhorn, Gottfried H.; Baums, Mike H. Biomechanical comparison of double-loaded suture anchors using arthroscopic Mason–Allen Stitches versus traditional transosseous suture technique and modified Mason–Allen stitches for rotator cuff repair. Clinical Biomechanics Jan2007, Vol. 22 Issue 1, p106. | Arthroscopic |
| Klinger, H.-M.; Koelling, S.; Baums, M. H.; Kahl, E.; Steckel, H.; Smith, M. M.; Schultz, W.; Miosge, N. Cell biological and biomechanical evaluation of two different fixation techniques for rotator cuff repair. Scandinavian Journal of Medicine & Science in Sports Jun2009, Vol. 19 Issue 3, p329. | Rotator Cuff |
| Klintberg, Ingrid Hultenheim; Gunnarsson, Ann-Christine; Svantesson, Ulla; Styf, Jorma; Karlsson, Jòn. Early loading in physiotherapy treatment after full-thickness rotator cuff repair: a prospective randomized pilot-study with a two-year follow-up. Clinical Rehabilitation Jul2009, Vol. 23 Issue 7, p622. | Rotator Cuff |
| Klintberg IH, Gunnarsson AC, Svantesson U, Styf J, Karlsson J. Early activation or a more protective regime after arthroscopic subacromial decompression -- a description of clinical changes with two different physiotherapy treatment protocols -- a prospecitve randomized pilot study with a two-year follow-up. 2008. Clinical Rehabilitation. | Arthroscopic |
| Koike K, Yamamoto Y, Suzuki N, Yamazaki R, Yoshikawa C, Takano F, Sugiura K, Inoue M. Efficacy of porcine placental extract on shoulder stiffness in climacteric women. Climacteric. 2013 Aug;16(4):447-52. doi:10.3109/13697137.2012.720622. Epub 2012 Nov 1. PubMed PMID: 23113540. | Placenta |
| Kösters, A.; Rieder, F.; Wiesinger, H.-P.; Dorn, U.; Hofstaedter, T.; Fink, C.; Müller, E.; Seynnes, O. R. Alpine Skiing With total knee ArthroPlasty ( ASWAP): effect on tendon properties. Scandinavian Journal of Medicine & Science in Sports Aug2015 Supplement, Vol. 25, p67. | Knee |
| Kraus, Natascha; Haas, Norbert; Scheibel, Markus; Gerhardt, Christian. Arthroscopically assisted stabilization of acute high-grade acromioclavicular joint separations in a coracoclavicular Double- TightRope technique: V-shaped versus parallel drill hole orientation. Archives of Orthopaedic & Trauma Surgery Oct2013, Vol. 133 Issue 10, p1431. | Arthroscopic |
| Kraus, Michael; von dem Berge, Stephanie; Schöll, Hendrik; Krischak, Gert; Gebhard, Florian. Integration of fluoroscopy-based guidance in orthopaedic trauma surgery – A prospective cohort study. Injury Nov2013, Vol. 44 Issue 11, p1486. | Observational |
| Kuhn, John E.; Dunn, Warren R.; Ma, Benjamin; Wright, Rick W.; Jones, Grant; Spencer, Edwin E.; Wolf, Brian; Safran, Marc; Spinder, Kurt P.; McCarty, Eric; Kelly, Brian; Holloway, Brian. Interobserver Agreement in the Classification of Rotator Cuff Tears. American Journal of Sports Medicine Mar 2007: Vol. 35 Issue 3. p. 437-441. | Rotator Cuff |
| Kuhn, John E. How Much Benefit do we get form rotator cuff repair. 2014. Journal of Bone and Joint Surgery. | Rotator Cuff |
| Kukkonen J, Juokainen A, Lehtinen J, Mattila KT, Tuominen EK, Kauko T, Aarimaa V. Treatment of nontraumatic rotator cuff tears: a randomized controlled trial with two years of clinical and imaging follow-up. 2015. Journal of Bone and Joint Surgery, American | Rotator Cuff |
| Kukkonen J, Juokainen A, Lehtinen J, Mattila KT, Tuominen EK, Kauko T, Aarimaa V. Treatment of nontraumatic rotator cuff tears: a randomized controlled trial with two years of clinical and imaging follow-up. 2015. Journal of Bone and Joint Surgery, American | Duplicate |
| Kweon, Christopher; Gagnier, Joel J.; Robbins, Christopher B.; Bedi, Asheesh; Carpenter, James E.; Miller, Bruce S. Surgical Versus Nonsurgical Management of Rotator Cuff Tears. American Journal of Sports Medicine Oct2015, Vol. 43 Issue 10, p2368. | Rotator Cuff |
| Kyoung Hwan Koh; Tae Kang Lim; Min Soo Shon; Young Eun Park; SeungWon Lee; Jae Chul Yoo. Effect of Immobilization without Passive Exercise After Rotator Cuff Repair: Randomized Clinical Trial Comparing Four and Eight Weeks of Immobilization. Journal of Bone & Joint Surgery, American Volume 3/19/2014, Vol. 96 Issue 6, pe44(1). | Rotator Cuff |
| Lakemeier, Stefan; Braun, Juliane; Efe, Turgay; Foelsch, Christian; Archontidou-Aprin, Eleni; Fuchs- Winkelmann, Susanne; Paletta, Juergen; Schofer, Markus. Expression of matrix metalloproteinases 1, 3, and 9 in differing extents of tendon retraction in the torn rotator cuff. Knee Surgery, Sports Traumatology, Arthroscopy Oct2011, Vol. 19 Issue 10, p1760. | Rotator Cuff |
| Lam, P.H.; Hansen, K.; Keighley, G; Hackett, L.; Murrell, G.A.C. A Randomized, Double-Blinded, Placebo-Controlled Clinical Trial Evaluating the Effictiveness of Daily Vibration After Arthroscopic Rotator Cuff Repair. 2015. American Journal of Sports Medicine. | Arthroscopic |
| Lapner, P. L.; Sabri, E.; Rakhra K. et al. A Multicenter randomized controlled trial comparing single-row with double-row fixation in arthroscopic rotator cuff repair. 2012. Journal of Bone and Joint Surgery, American. | Arthroscopic |
| Lapner, P. L.; Sabri, E.; Rakhra, K.; Bell, K.; Athwal, G. S. Healing rates and subscapularis fatty infiltration after lesser tuberosity osteotomy versus subscapularis peel for exposure during shoulder arthroplasty. 2013. Journal of Shoulder and Elbow Surgery. | Non-RCT |
| Lapner, Peter L. C.; Sabri, Etham; Rakhra, Kawan; Bell, Kimberly; Athwal, George S. Comparison of Lesser Tuberosity Osteotomy to Subscapularis Peel in Shoulder Arthroplasty. 2012 Journal of Bone and Joint Surgery, American | Duplicate |
| Laudner, Kevin G.; Metz, Betsy; Thomas, David Q. Anterior Glenohumeral Laxity and Stiffness After a Shoulder- Strengthening Program in Collegiate Cheerleaders. Journal of Athletic Training (Allen Press) Jan/Feb2013, Vol. 48 Issue 1, p25. | Physical Therapy |
| Laudner, Kevin G.; Williams, Jeffrey G. The relationship between latissimus dorsi stiffness and altered scapular kinematics among asymptomatic collegiate swimmers. Physical Therapy in Sport 2013, Vol. 14 Issue 1, p50. | Shoulder, Not Arthroplasty |
| Launonen, Antti P; Lepola, Vesa; Flinkkil, Tapio; Laitinen, Minna; Paavola, Mika; Malmivaara, Antti, Acta. Treatment of proximal humerus fractures in the elderly. Orthopaedica Jun2015, Vol. 86 Issue 3, p280 | Systematic Review |
| Lee, Hyo-Jin; Kim, Yang-Soo; Ok, Ji-Hoon; Lee, Yun-Kyoung; Ha, Michelle. Effect of a single subacromial prednisolone injection in acute rotator cuff tears in a rat model. Knee Surgery, Sports Traumatology, Arthroscopy Feb2015, Vol. 23 Issue 2, p555. | Animal |
| Lee, Sang; Park, Sang-Eun; Nam, Yong-Seok; Han, Seung-Ho; Lee, Kwang-Jin; Kwon, Min-Jeong; Ji, Jong-Hun; Choi, Syung-Kyun; Park, Jang-Su. Analgesic effectiveness of nerve block in shoulder arthroscopy: comparison between interscalene, suprascapular and axillary nerve blocks. Knee Surgery, Sports Traumatology, Arthroscopy Dec2012, Vol. 20 Issue 12, p2573. | Arthroscopic |
| Lee, Jae; Yoo, Yon-Sik; Hwang, Jung-Taek; Kim, Do-Young; Jeon, Seong-Jae; Hwang, Sung; Jang, Ji. Efficacy of direct arthroscopy-guided suprascapular nerve block after arthroscopic rotator cuff repair: a prospective randomized study.  Knee Surgery, Sports Traumatology, Arthroscopy Feb2015, Vol. 23 Issue 2, p562. | Rotator Cuff |
| Lee, Donald H. Platelet-Rich Plasma: Is It Ready for Prime Time? 2012. Journal of Bone and Joint Surgery. | PRP Injection |
| Lenters, Tim R.; Franta, Amy K.; Wolf, Fredric M.; Leopold, Seth S.; Matsen III, Frederick A. arthroscopic Compared with Openn repairs for recurrent anterior shoulder instability. 2007. Journal of Bone and Joint Surgery. | Arthroscopic |
| Lewis, Jeremy S. A specific exercise program for patients with subacromial impingement syndrome can improve function and reduce the need for surgery. Journal of Physiotherapy (Elsevier) Jun2012, Vol. 58 Issue 2, p127 | Rotator Cuff |
| Liavaag, Sigurd; Stiris, Morten Georg; Lindland, Elisabeth Stokke; Enger, Martine; Svenningsen, Svein; Brox, Jens Ivar. Do Bankart lesions heal better in shoulders immobilized in external rotation? Acta Orthopaedica Oct2009, Vol. 80 Issue 5, p579. | Arthroscopic |
| Lill, Helmut; Katthagen, Christoph; Hertel, Alexandra; Gille, Justus; Voigt, Christine. All-arthroscopic intramedullary nailing of 2- and 3-part proximal humeral fractures: a new arthroscopic technique and preliminary results.  Archives of Orthopaedic & Trauma Surgery May2012, Vol. 132 Issue 5, p641. | Arthroscopic |
| Lindenhovius, A.L.C.; Doornbery, J.N.; Brouwer, K.M.; et al. A prospective randomized controlled trial of dynamic versus static progressive elbow splinging for posttraumatic elbow stiffness. 2012. Journal of Bone and Joint Surgery, American. | Elbow |
| Lisowski JK, Oyama S, Hibberd EE. Biceps brachii does not play an active role in humeral movement. 2014. The Journal of the Practicing Clinician. | Biceps |
| Litchfield, Robert. Progressive Strengthening Exercises for Subacromial Impingement Syndrome. Clinical Journal of Sport Medicine Jan2013, Vol. 23 Issue 1, p86. | Rotator Cuff |
| Lo, Ian K. Y.; Litchfield, Robert B.; Griffin, Sharon; Faber, Ken; Patterson, Stuart D.; Kirkley, Alexandra. QUALITY-OF-LIFE OUTCOME FOLLOWING HEMIARTHROPLASTY OR TOTAL SHOULDER ARTHROPLASTY IN PATIENTS WITH OSTEOARTHRITIS. 2005. Journal of Bone and Joint Surgery. | Duplicate |
| Lorbach, Olaf; Baums, Mike; Kostuj, Tanja; Pauly, Stephan; Scheibe, Markus; Carr, Andrew; Zargar, Nasim; Saccomanno, Maristella; Milano, Giuseppe. Advances in biology and mechanics of rotator cuff repair. 2015 Knee Surgery, Sports Traumatology, Arthroscopy. | Rotator Cuff |
| Lovric, Vedran; Ledger, Michael; Goldberg, Jerome; Harper, Wade; Bertollo, Nicky; Pelletier, Matthew; Oliver, Rema; Yu, Yan; Walsh, William. The effects of Low-intensity Pulsed Ultrasound on tendon-bone healing in a transosseous-equivalent sheep rotator cuff model. Knee Surgery, Sports Traumatology, Arthroscopy Feb2013, Vol. 21 Issue 2, p466. | Animal |
| Lozano-Calderón, Santiago A.; Souer, Sebastiaan; Mudgal, Chaitanya; Jupiter, Jesse B.; Ring, David. Wrist Mobilization Following Volar Plate Fixation of Fractures of the Distal Part of the Radius. Journal of Bone & Joint Surgery, American Volume Jun2008, Vol. 90-A Issue 6, p1297. | Radius |
| Lu Y, Zhang Q, Zhu Y, Jiang C. Is radiofrequency treatment effective for shoulder impingement syndrome? A prospective randomized controlled study. J Shoulder Elbow Surg. 2013 Nov;22(11):1488-94. doi: 10.1016/j.jse.2013.06.006. Epub 2013 Aug 30. PubMed PMID: 23994459. | Arthroscopic |
| Lu Y, Li Y, Li F, Jiang C. Perspective randomized control study on different NSAIDs drugs after rotator cuff repair. 2015. Zhonghua Yi Xue Za Zhi. | Pharmacology |
| Ma, C. Benjamin; Comerford, Lyn; Wilson, Joseph; Puttlitz, Christian M. BIOMECHANICAL EVALUATION OF ARTHROSCOPIC ROTATOR CUFF REPAIRS: DOUBLE-ROW COMPARED WITH SINGLE-ROW FIXATION. ournal of Bone & Joint Surgery, American Volume Feb2006, Vol. 88-A Issue 2, p403. | Arthroscopic |
| MacDonald P, McRae S, Leiter J, Mascarenhas R, Lapner P. Arthroscopic rotator cuff repair with and without acromioplasty in the treatment of full-thickness rotator cuff tears: a multicenter, randomized controlled trial. 2011. Journal of Bone and Joint Surgery, American | Rotator Cuff |
| MacDonald P, McRae S, Leiter J, Mascarenhas R, Lapner P. Arthroscopic rotator cuff repair with and without acromioplasty in the treatment of full-thickness rotator cuff tears: a multicenter, randomized controlled trial. 2011. Journal of Bone and Joint Surgery, American | Duplicate |
| Mahabier, Kiran C.; Vogels, Lucas M.M.; Punt, Bas J.; Roukema, Gert R.; Patka, Peter; Van Lieshout, Esther M.M. Humeral shaft fractures: Retrospective results of non-operative and operative treatment of 186 patients . Injury Apr2013, Vol. 44 Issue 4, p427. | Observational |
| Mahadeva, D.; Dias, R.G.; Deshpande, S.V.; Datta, A.; Dhillon, S.S.; Simons, A.W. The reliability and reproducibility of the Neer classification system – Digital radiography (PACS) improves agreement. Injury Apr2011, Vol. 42 Issue 4, p339 | Radiology |
| Mahowald ML, Krug HE, Singh JA, Dykstra D. Intra-articular Botulinum Toxin Type A: a new approach to treat arthritis joint pain. Toxicon. 2009 Oct;54(5):658-67. doi: 10.1016/j.toxicon.2009.03.028. Epub 2009 Apr 5. PubMed PMID: 19351542. | Pharmacology |
| Malavolta, E.A,; Gracitelli, M.E.C.; Ferreira Neto, A.A.; Assuncao, J.H.; Bordalo-Rodrigues, M.; de Camargo, O.P. Platelet-Rich plasma in rotator cuff repair: a prospective randomized study. 2014. American Journal of Sports Medicine. | Rotator Cuff |
| Marquardt, Bjoern; Hurschler, Christof; Schneppendahl, Johannes; Witt, Kai-Axel; Poetzl, Wolfgang; Steinbeck, Joern. Quantitative Assessment of Glenohumeral Translation After Anterior Shoulder Dislocation and Subsequent Arthroscopic Bankart Repair. American Journal of Sports Medicine Nov 2006: Vol. 34 Issue 11. p. 1756-1762. | Arthroscopic |
| Marshall-McKenna, Rebecca; Paul, Lorna; McFadyen, Angus K.; Gilmartin, Alexandra; Armstrong, Anne; Rice, Ann Marie; McIlroy, Pauline. Myofascial release for women undergoing radiotherapy for breast cancer: a pilot study. 2014. European Journal of Physiotherapy. | Cancer |
| Martin, Kevin D.; Belmont, Philip J.; Schoenfeld, Andrew J.; Todd, Michael; Cameron, Kenneth L.; Owens, Brett D. Arthroscopic Basic Task Performance in Shoulder Simulator Model Correlates with Similar Task Performance in Cadavers. Journal of Bone & Joint Surgery, American Volume 11/2/2011, Vol. 93-A Issue 21, p2047. | Cadaver |
| Martin, Nathan; Martin, Giselle. Casey's Course. Australian Tennis Magazine: Asia & the Pacific Jan2010, Vol. 35 Issue 1, p57. | Commentary |
| Mazzocca, Augustus D.; Millett, Peter J.; Guanche, Carlos A.; Santangelo, Stephen A.; Arciero, Robert A. Arthroscopic Single-Row Versus Double-Row Suture Anchor Rotator Cuff Repair. American Journal of Sports Medicine Dec 2005: Vol. 33 Issue 12. p. 1861-1868 | Arthroscopic |
| Mazzocca, Augustus D.; Santangelo, Stephen A.; Johnson, Sean T.; Rios, Cliffors G.; Dumonski, Mark L.; Arciero, Robert A. A Biomechanical Evaluation of an Anatomical Coracoclavicular Ligament Reconstruction.  American Journal of Sports Medicine Feb 2006: Vol. 34 Issue 2. p. 236-246 | Shoulder, Not Arthroplasty |
| McCormack, Jr., Michael A.; Lindenfeld, Thomas N.; Barber-Westin, Sue D. Comparing Two Devices Used to Regain Full Range of Motion Following Arthroscopic Subacromial Decompression for Shoulder Impingement. Athletic Training & Sports Health Care: The Journal for the Practicing Clinician Jan/Feb2012, Vol. 4 Issue 1, p21. | Arthroscopic |
| McFadyen, I.; Field, J.; McCann, P.; Ward, J.; Nicol, S.; Curwen, C. Should unstable extra-articular distal radial fractures be treated with fixed-angle volar-locked plates or percutaneous Kirschner wires? A prospective randomised controlled trial. 2011. Injury. | Radius |
| McKee MD, Veillette CJ, Hall JA, Schemitsch EH, Wild LM, McCormack R et al. A multicenter, prospective, randomized, controlled trial of open reduction--internal fixation versus total elbow arthroplasty for displaced intra-articular distal humeral fractures in elderly patients. 2009. Journal of Shoulder and Elbow Surgery. | Elbow |
| Mesiha, Mena M.; Derwin, Kathleen A.; Sibole, Scott C.; Erdemir, Ahmet; McCarron, Jesse A. The Biomechanical Relevance of Anterior Rotator Cuff Cable Tears in a Cadaveric Shoulder Model. Journal of Bone & Joint Surgery, American Volume 10/16/2013, Vol. 95-A Issue 20, p1817. | Cadaver |
| Michener, Lori; Subasi Yesilyaprak, Sevgi; Seitz, Amee; Timmons, Mark; Walsworth, Matthew. Supraspinatus tendon and subacromial space parameters measured on ultrasonographic imaging in subacromial impingement syndrome.  Knee Surgery, Sports Traumatology, Arthroscopy Feb2015, Vol. 23 Issue 2, p363. | Radio |
| Michlitsch, Michael G.; Adamson, Gregory J.; Pink, Marilyn; Estess, Allyson; Shankwiler, James A.; Lee, Thay Q. Biomechanical Comparison of a Modified Weaver-Dunn and a Free- Tissue Graft Reconstruction of the Acromioclavicular Joint Complex. American Journal of Sports Medicine Jun2010, Vol. 38 Issue 6, p1196. | AC joint |
| Milano, Giuseppe; Grasso, Andrea; Santagada, Domenico A.; Saccomanno, Maristella F.; Deriu, Laura; Fabbriciani, Carlo. Comparison between metal and biodegradable suture anchors in the arthroscopic treatment of traumatic anterior shoulder instability: a prospective randomized study. Knee Surgery, Sports Traumatology, Arthroscopy Dec2010, Vol. 18 Issue 12, p1785. | Arthroscopic |
| Milano G, Saccomanno MF, Careri S, Taccardo G, De Vitis R, Fabbriciani C. Efficacy of marrow-stimulating technique in arthroscopic rotator cuff repair: a prospective randomized study. 2013. Arthroscopy. | Rotator Cuff |
| Miller, Bruse S. Commentary on an article by Sigurd Liavaag, MD, et al.: "Immobilization in external rotation after primary shoulder dislocation did not reduce the risk of recurrence. A randomized controlled trial". 2011. Journal of Bone and Joint Surgery. | Commentary |
| MITSUNORI TOKUDA; KOJI SHOMOTO; YASUHARU TOMITA, Rigakuryoho Kagaku. Effects of Transcutaneous Electrical Nerve Stimulation in Postoperative Shoulder Patients--Where is the Best Electrode Placement ? 2012, Vol. 27 Issue 5, p565. | Electrical Stimulation |
| Mohtadi, Nick. Accuracy of Subacromial Injestions. (Abstract). Clinical Journal of Sport Medicine May 2007: Vol. 17 Issue 3. p. 226-227. | Abstract |
| Mohtadi, N.G.; Hollinshead, R.M.; Sasyniuk, T.M.; fletcher, J.A.; Chan, D.S.; Li, F.X. A Randomized Clinical Trail Comparing Mini-open With Open Rotator Cuff Repair: Two-year Outcomes. (Abstract). Clinical Journal of Sport Medicine Mar 2006: Vol. 16 Issue 2. p. 182. | Abstract |
| Mohtadi, Nick. Exercises or Arthroscopic Decompression for Subacromial Impingement? Clinical Journal of Sport Medicine Mar 2006: Vol. 16 Issue 2. p. 193-194. | Arthroscopic |
| Mohtadi, Nicholas G.; Hollinshead, Robert M.; Sasyniuk, Treny M.; Fletcher, Jennifer A.; Chan, Denise S.; Li, Feng X. A Randomized Clinical Trial Comparing Open to Arthroscopic Acromioplasty With Mini-Open Rotator Cuff Repair for Full-Thickness Rotator Cuff Tears. American Journal of Sports Medicine Jun2008, Vol. 36 Issue 6, p1043. | Duplicate |
| Mohtadi NG, Hollinshead RM, Sasyniuk TM, Fletcher JA, Chan DS, Li FX. A randomized clinical trial comparing open to arthroscopic acromioplasty with mini-open rotator cuff repair for full-thickness rotator cuff tears: disease-specific quality of life outcome at an average 2-year follow-up. 2008. American Journal of Sports Medicine. | Duplicate |
| Mohtadi NG, Hollinshead RM, Sasyniuk TM, Fletcher JA, Chan DS, Li FX. A randomized clinical trial comparing open to arthroscopic acromioplasty with mini-open rotator cuff repair for full-thickness rotator cuff tears: disease-specific quality of life outcome at an average 2-year follow-up. 2008. American Journal of Sports Medicine. | Arthroscopic |
| Monk, A. Paul; Garfjeld Roberts, Patrick; Logishetty, Kartik; Price, Andrew J.; Kulkarni, Rohit; Rangan, Amar; Rees, Jonathan L. Evidence in managing traumatic anterior shoulder instability: a scoping review. British Journal of Sports Medicine Mar2015, Vol. 49 Issue 5, p1. | Arthroscopic |
| Moosmayer, Stefan; Lund, Gerty; Seljom, Unni S.; Haldorsen, Benjamin; Svege, Ida C.; Hennig, Toril; Pripp, Are H.; Smith, Hans-Jørgen. Tendon Repair Compared with Physiotherapy in the Treatment of Rotator Cuff Tears. Journal of Bone & Joint Surgery, American Volume 9/17/2014, Vol. 96 Issue 18, p1504. | Rotator Cuff |
| Mousavi, Sayed Javad; Parnianpour, Mohamad; Abedi, Mohsen; Askary-Ashtiani, Ahmadreza; Karimi, Abdolkarim; Khorsandi, Aliakbar; Mehdian, Hossein. Cultural adaptation and validation of the Persian version of the Disabilities of the Arm, Shoulder and Hand (DASH) outcome measure. Clinical Rehabilitation Aug2008, Vol. 22 Issue 8, p749. | Shoulder, Not Arthroplasty |
| Myers, Thomas H.; Zemanovic, Jason R.; Andrews, James R. The Resisted Supination External Rotation Test A New Test for the Diagnosis of Superior Labral Anterior Posterior Lesions. American Journal of Sports Medicine Sept 2005: Vol. 33 Issue 9. p. 1315-1320 | Shoulder, Not Arthroplasty |
| n/a | Abstract |
| n/a | Abstract |
| n/a | Abstract |
| n/a | Abstract |
| n/a | Abstract |
| n/a | Abstract |
| n/a | Commentary |
| n/a | Physical Therapy |
| n/a | Rotator Cuff |
| Namdari, Surena; Donegan, Ryan P.; Chamberlain, Aaron M.; Galatz, Leesa M.; Yamaguchi, Ken; Keener, Jay D. Factors Affecting Outcome After Structural Failure of Repaired Rotator Cuff Tears. Journal of Bone & Joint Surgery, American Volume 1/15/2014, Vol. 96 Issue 2, p99. | Rotator Cuff |
| Neviaser, A.; Braman, J,; Parsons, B. et al. What's new in shoulder and elbow surgery. 2013. Journal of Bone and Joint Surgery. | Elbow |
| Neviaser, Andrew S.; Hannafin, Jo A. Adhesive Capsulitis. 2010. American Journal of Sports Medicine. | Capsulitis |
| Nguyen D, Ferreira LM, Brownhill JR, King GJW, Drosdowech DS, Faber KJ, Johnson JA. Improved accuracy of computer assisted glenoid implantation in total shoulder arthroplasty: An in-vitro randomized controlled trial. 2009. Journal of Shoulder and Elbow Surgery. | In Vitro |
| Sebastiaan Souer, J.; Buijze, Geert; Ring, David. A Prospective Randomized Controlled Trial Comparing Occupational Therapy with Independent Exercises After Volar Plate Fixation of a Fracture of the Distal Part of the Radius. Journal of Bone & Joint Surgery, American Volume 10/5/2011, Vol. 93-A Issue 19, p1761. | Arthroscopic |
| Nho, Shane J.; Frank, Rachel M.; van Thiel, Geoffrey S.; Fan Chia Wang; Wang, Vincent M.; Provencher, Matthew T.; Verma, Nikhil N. A Biomechanical Analysis of Anterior Bankart Repair Using Suture Anchors.  American Journal of Sports Medicine Jul2010, Vol. 38 Issue 7, p1405. | Arthroscopic |
| Nho, Shane J.; Frank, Rachel M.; van Thiel, Geoffrey S.; Fan Chia Wang; Wang, Vincent M.; Provencher, Matthew T.; Verma, Nikhil N. A Biomechanical Analysis of Shoulder Stabilization. American Journal of Sports Medicine Jul2010, Vol. 38 Issue 7, p1413. | Shoulder, Not Arthroplasty |
| Nuttall, D.; Haines, J. F.; Trail, I. I. Radiostereographic analysis of a shoulder surface replacement: does hydroxyapatite have a place? 2014. Bone and Joint Journal. | Article Not Found |
| NYFFELER, RICHARD W.; WERNER, CLÉMENT M. L.; SUKTHANKAR, ATUL; SCHMID, MARIUS R.; GERBER, CHRISTIAN. ASSOCIATION OF A LARGE LATERAL EXTENSION OF THE ACROMION WITH ROTATOR CUFF TEARS. Journal of Bone & Joint Surgery, American Volume Apr2006, Vol. 88-A Issue 4, p800. | Rotator Cuff |
| Oh JH, Kim JY, Choi JH, Park SM. Is arthroscopic distal clavicle resection necessary for patients with radiological acromioclavicular joint arthritis and rotator cuff tears? A prospective randomized comparative study. 2014. American Journal of Sports Medicine. | Rotator Cuff |
| Oh, J.H.; Kim, J.Y. Choi, J.H.; Park, S.M. Is arthroscopic distal clavicle resection necessary for patients with radiological acromioclavicular joint arthristis and rotator cuff tears? A prospective Randomized Comparative Study. 2014. American Journal of Sports Medicine. | Duplicate |
| OHTSUKI, KEISUKE; ISHIKURA, TAKASHI, Rigakuryoho Kagaku. A Clinical Anatomical Physical Therapy Approach for Frozen Shoulder Joint Patients.  2010, Vol. 25 Issue 4, p493. | Physical Therapy |
| Ok JH, Kim YS, Kim JM, Yoon KS. A new technique of arthroscopic fixation using double anchors for SLAP lesions. 2012. Knee Surgery, Sports Traumatology, Arthroscopy. | Arthroscopic |
| Osti L, Buono AD, Maffulli N. Microfractures at the rotator cuff footprint: a randomised controlled study. 2013. International Orthopedics. | Rotator Cuff |
| Osti L, Buono AD, Maffulli N. Pulsed electromagnetic fields after rotator cuff repair: a randomized, controlled study. 2015. Orthopedics. | Rotator Cuff |
| Pagonis, Thomas; Ditsios, Konstantinos; Toli, Paraskevi; Givissis, Panagiotis; Christodoulou, Anastasios. Improved Corticosteroid treatment of recalcitrant de quervain tenosynovitis with a novel 4-point injection technique. 2011. American Journal of Sports Medicine. | Wrist/Hand |
| Pan, Xiaoyun; Ye, Luyou; Liu, Zhongtang; Wen, Hong; Hu, Yuezheng; Xu, Xinxian. Effect of irrigation fluid temperature on core body temperature and inflammatory response during arthroscopic shoulder surgery. Archives of Orthopaedic & Trauma Surgery Aug2015, Vol. 135 Issue 8, p1131. | Arthroscopic |
| Park, Ji Soon; McGarry, Michelle H.; Campbell, Sean T.; Seo, Hyuk Jun; Lee, Yeon Soo; Kim, Sae Hoon; Lee, Thay Q.; Oh, Joo Han. The Optimum Tension for Bridging Sutures in Transosseous- Equivalent Rotator Cuff Repair. American Journal of Sports Medicine Sep2015, Vol. 43 Issue 9, p2118. | Rotator Cuff |
| Park YB, Koh KH, Shon MS, Park YE, Yoo JC. Arthroscopic distal clavicle resection in symptomatic acromioclavicular joint arthritis combined with rotator cuff tear: a prospective randomized trial. 2015 American Journal of Sports Medicine. | Rotator Cuff |
| Park YB, Koh KH, Shon MS, Park YE, Yoo JC. Arthroscopic distal clavicle resection in symptomatic acromioclavicular joint arthritis combined with rotator cuff tear: a prospective randomized trial. 2015 American Journal of Sports Medicine. | Duplicate |
| Parsons BO, Getz CL, Ramsey ML. What's new in shoulder and elbow surgery? 2012. Journal of Bone and Joint Surgery. | Literature Review |
| Patel, Rahul V.; Leith, Jordan; Robinson, C. Michael; Jenkins, Paul J. Primary Arthroscopic Stabilization for a First-Time Anterior Dislocation of the Shoulder. Journal of Bone & Joint Surgery, American Volume Aug2008, Vol. 90-A Issue 8, p1786. | Arthroscopic |
| Pauly, Stephan; Gerhardt, Christian; Haas, Norbert; Scheibel, Markus. Prevalence of concomitant intraarticular lesions in patients treated operatively for high-grade acromioclavicular joint separations. Knee Surgery, Sports Traumatology, Arthroscopy Jul2009, Vol. 17 Issue 5, p513. | AC joint |
| Piitulainen, K.; Hakkinen, A.; Salo, P.; Kautiainen, H.; Ylinen, J. Does adding a 12-month exercise programme to usual care after a rotator cuff repair effect disability and quality of life at 12 months? A randomized controlled trial. 2015. Clinical Rehabilitation. | Rotator Cuff |
| Plausinis, Derek; Bravman, Jonathan T.; Heywood, Christian; Kummer, Frederick J.; Kwon, Young W.; Jazrawi, Laith M. Arthroscopic Rotator Interval Closure Effect of Sutures on Glenohumeral Motion and Anterior-Posterior Translation. American Journal of Sports Medicine Oct 2006: Vol. 34 Issue 10. p. 1656-1661. | Arthroscopic |
| Ponce, Brent A.; Hosemann, Chad D.; Raghava, Parthasarathy; Tate, Janet P.; Eberhardt, Alan W.; Lafosse, Laurent. Biomechanical Evaluation of 3 Arthroscopic Self-Cinching Stitches for Shoulder Arthroscopy. American Journal of Sports Medicine 01/01/2011, Vol. 39 Issue 1, p188. | Arthroscopic |
| Ponce, Brent A.; Rosenzweig, Seth D.; Thompson, Kevin J.; Tokish, John. Sequential Volume Reduction With Capsular Plications. American Journal of Sports Medicine 03/01/2011, Vol. 39 Issue 3, p526. | Arthroscopic |
| Ponce, Brent A.; Jennings, Jonathan K.; Clay, Terry B.; May, Mathew B.; Huisingh, Carrie; Sheppard, Evan D. Telementoring: Use of Augmented Reality in Orthopaedic Education.  Journal of Bone & Joint Surgery, American Volume 5/21/2014, Vol. 96 Issue 10, pe84-1. | Educational |
| Poon PC.; Chou J.; Young SW.; Astley T. A comparison of concentric and eccentric glenospheres in reverse shoulder arthroplasty: a randomized controlled trial. 2014. The Journal of Bone and Joint Surgery. | Duplicate |
| Rahme, H.; Mattsson, P.; Wikblad, L.; Nowak, J.; Larsson, S. Stability of cemented in-line pegged glenoid compared with keeled glenoid components in total shoulder arthroplasty. 2009. Journal of Bone and Joint Surgery - Series A. | Duplicate |
| Raiss, Patric; Baumann, Florian; Akbar, Michael; Rickert, Markus; Loew, Markus. Open screw fixation of large anterior glenoid rim fractures: mid- and long-term results in 29 patients.  Knee Surgery, Sports Traumatology, Arthroscopy Feb2009, Vol. 17 Issue 2, p195. | Shoulder, Not Arthroplasty |
| Ranalletta, Maximiliano; Rossi, Luciano A.; Piuzzi, Nicolás S.; Bertona, Agustin; Bongiovanni, Santiago L.; Maignon, Gaston. Return to Sports After Plate Fixation of Displaced Midshaft Clavicular Fractures in Athletes.  American Journal of Sports Medicine Mar2015, Vol. 43 Issue 3, p565. | Clavicle |
| Rangan A.; Handoll H.; Brealey S.; Jefferson L.; Keding A.; Martin B.C. ; Goodchild L.; Chuang L. H. ; Hewitt C.; Torgerson D.; Krieg, James C. Surgical and nonsurgical treatment produced similar outcomes for proximal humeral fractures. 2015 Journal of Bone and Joint Surgery | Article Not Found |
| Reid MJ, Booth G, Khan RJ, Janes G. Patellar eversion during total knee replacement: a prospective, randomized trial. 2014. Journal of Bone and Joint Surgery, American | Knee |
| Reinold, Michael M.; Macrina, Leonard C.; WiIk, Kevin E.; Dugas, Jeffrey R.; Cain, E. Lyle; Andrews, James R. The Effect of Neuromuscular Electrical Stimulation of the Infraspinatus on Shoulder External Rotation Force Production After Rotator Cuff Repair Surgery. American Journal of Sports Medicine Dec2008, Vol. 36 Issue 12, p2317. | Rotator Cuff |
| Rempel D, Lee DL, Dawson K, Loomer P. The effects of periodontal curette handle weight and diameter on arm pain: a four-month randomized controlled trial. J Am Dent Assoc. 2012 Oct;143(10):1105-13. PubMed PMID: 23024308. | Upper Extremity, Not Shoulder |
| Rigsby, Ruel; Sitler, Michael; Kelly, John D. Subscapularis Tendon Integrity: An Examination of Shoulder Index Tests. Journal of Athletic Training (National Athletic Trainers' Association) Jul/Aug2010, Vol. 45 Issue 4, p404 | Rotator Cuff |
| Rios, Daniel; Jansson, Kyle; Martetschläger, Frank; Boykin, Robert; Millett, Peter; Wijdicks, Coen. Normal curvature of glenoid surface can be restored when performing an inlay osteochondral allograft: an anatomic computed tomographic comparison. Knee Surgery, Sports Traumatology, Arthroscopy Feb2014, Vol. 22 Issue 2, p442. | Arthroscopic |
| Robinson CM, Jenkins PJ, White TO, Ker A, Will E. Primary arthroscopic stabilization for a first-time anterior dislocation of the shoulder: a randomized, double-blind trial. 2008. Journal of Bone and Joint Surgery. | Arthroscopic |
| Rodeo, Scott A.; Delos, Demetris; Williams, Riley J.; Adler, Ronald S.; Pearle, Andrew; Warren, Russell F. The effect of plately-rich fibrin matrix on rotator cuff tendon healing: a prospective, randomized clinical study. 2012. American Journal of Sports Medicine. | Rotator Cuff |
| Rozental, Tamara D.; Blazar, Philip E.; Franko, Orrin I.; Chacko, Aron T.; Earp, Brandon E.; Day, Charles S. Functional Outcomes for Unstable Distal Radial Fractures Treated with Open Reduction and Internal Fixation or Closed Reduction and Percutaneous Fixation: A Prospective Randomized Trial.  Journal of Bone & Joint Surgery, American Volume Aug2009, Vol. 91-A Issue 8, p1837. | Radius |
| Sabeti, M.; Schmidt, M.; Ziai, P.; Graf, A.; Nemecek, E.; Schueller-Weidekamm, C. The intraoperative use of ultrasound facilitates significantly the arthroscopic debridement of calcific rotator cuff tendinitis.  Archives of Orthopaedic & Trauma Surgery May2014, Vol. 134 Issue 5, p651. | Duplicate |
| Sabeti-Aschraf, Manuel; Lemmerhofer, B.; Lang, S.; Schmidt, M.; Funovics, P. T.; Ziai, P.; Frenzel, S.; Kolb, A.; Graf, A.; Schueller-Weidekamm, C. Ultrasound guidance improves the accuracy of the acromioclavicular joint infiltration: a prospective randomized study.  Knee Surgery, Sports Traumatology, Arthroscopy Feb2011, Vol. 19 Issue 2, p292. | AC joint |
| Sabeti, M.; Schmidt, M.; Ziai, P. et al. The intraoperative use of ultrasound facilitates significantly the arthroscopic debridement of calcific rotator cuff tendinitis. 2014. Archives of Orthopaedic and Trauma. | Arthroscopic |
| Sadoghi, Patrick; Vavken, Julia; Leithner, Andreas; Vavken, Patrick. Benefit of intraoperative navigation on glenoid component positioning during total shoulder arthroplasty.  Archives of Orthopaedic & Trauma Surgery Jan2015, Vol. 135 Issue 1, p41. | Article Not Found |
| Sagen, A.; Kaaresen, R.; Sandvik, L.; Thune, I.; Risbery, M.A. Upper limb physical function and adverse effects after breat cancer surgery: a prospective 2.5-year follow-up study and preoperative measures. 2014. Archives of Physical Medicine and Rehabilitation. | Cancer |
| Salomonsson, Björn; Abbaszadegan, Hassan; Revay, Suzanne; Lillkrona, Ulf. The Bankart repair versus the Putti-Platt procedure. Acta Orthopaedica Jun2009, Vol. 80 Issue 3, p351. | Arthroscopic |
| Saltychev, M.; Aarimaa, V.; Virolainen, P.; Laimi, K. Conservative treatment or surgery for shoulder impingement: a systematic review and meta-analysis. 2015. Disability and Rehabilitation, | Systematic Review |
| Sang-in Park; Yong-Kyu Choi; Jung-Ho Lee; Young-Min Kim. Effects of shoudler stabilization exercise on pain and functional recovery of shoulder impingement syndrome patients. 2013. Journal of Physical Therapy Science. | Impingement Syndrome |
| Sauers, Eric L. Effectiveness of rehabilitation for patients with subacromial impingement syndrome. 2005. Journal of Athletic Training. | Impingement Syndrome |
| Saltzman, Matthew D.; Nuber, Gordon W.; Gryzlo, Stephen M.; Marecek, Geoffrey S.; Koh, Jason L. Efficacy of Surgical Preparation Solutions in Shoulder Surgery. Journal of Bone & Joint Surgery, American Volume Aug2009, Vol. 91-A Issue 8, p1949. | Shoulder, Not Arthroplasty |
| Sayegh, Fares E.; Kenanidis, Eustathios I.; Papavasiiou, Kyriakos A.; Potoupnis, Michael E.; Kirkos, John M.; Kapetanos, George A. Reduction of acuted andterior dislocations: a prospective randomized study comparing a new techniquie withe the Hippocratic and Kocher Methods. 2009. Journal of Bone and Joint Surgery. | Dislocation |
| Scheibel, Markus; Nikkulka, Constanze; Dick, Anton; Schroeder, Ralf Juergen; Popp, Ariane Gerber; Haas, Norbert P. Structural Integrity and Clinical Function of the Subscapularis Musculotendinous Unit After Arthroscopic and Open Shoulder Stabilization. American Journal of Sports Medicine July 2007: Vol. 35 Issue 7. p. 1153-1161. | Arthroscopic |
| Sebastiaan SJ, Buijze G, Ring D. A prospective randomized controlled trial comparing occupational therapy with independent exercises after volar plate fixation of a fracture of the distal part of the radius. 2011. Journal of Bone and Joint Surgery. | Radius |
| Sevivas, N.; Serra, S.; Portugal, R.; Teixeira, F.; Carvalho, M.; Silva, N.; Espregueira-Mendes, J.; Sousa, N.; Salgado, A. Animal model for chronic massive rotator cuff tear: behavioural and histologic analysis.  Knee Surgery, Sports Traumatology, Arthroscopy Feb2015, Vol. 23 Issue 2, p608. | Animal |
| Scheibel, Markus; Brown, Anna; Woertler, Klaus; Imhoff, Andreas. Preliminary results after rotator cuff reconstruction augmented with an autologous periosteal flap. Knee Surgery, Sports Traumatology, Arthroscopy Mar2007, Vol. 15 Issue 3, p305. | Rotator Cuff |
| Scheibel, Markus; Tsynman, Alexander; Magosch, Petra; Schroeder, Ralf Juergen; Habermeyer, Peter. Scheibel, Markus; Tsynman, Alexander; Magosch, Petra; Schroeder, Ralf Juergen; Habermeyer, Peter. American Journal of Sports Medicine Oct 2006: Vol. 34 Issue 10. p. 1586-1593 | Rotator Cuff |
| Shen C, Tang ZH, Hu JZ, Zou GY, Xiao RC, Yan DX. Does immobilization after arthroscopic rotator cuff repair increase tendon healing? A systematic review and meta-analysis. 2014. Archives of Orthopedic and Trauma Surgery. | Arthroscopic |
| Sherman, Seth L.; Lin, Emery C.; Verma, Nikhil N.; Mather, Richard C.; Gregory, James M.; Dishkin, Justin; Harwood, Daniel P.; Wang, Vincent M.; Shewman, Elizabeth F.; Cole, Brian J.; Romeo, Anthony A. Biomechanical Analysis of the Pectoralis Major Tendon and Comparison of Techniques for Tendo-osseous Repair. American Journal of Sports Medicine Aug2012, Vol. 40 Issue 8, p1887. | Pectoralis Major |
| Shi Q, MacDermid J, Grewak R, King GJ. Predictors of functional outcome change 18 months after anterior ulnar transposition. 2012. ARchives of Physical Medicine and Rehablitation. | Ulnar Nerve |
| Shu, Beatrice; Johnston, Tyler; Lindsey, Derek P.; McAdams, Timothy R. Biomechanical Evaluation of a Novel Reverse Coracoacromial Ligament Reconstruction for Acromioclavicular Joint Separation. American Journal of Sports Medicine Feb2012, Vol. 40 Issue 2, p440. | AC joint |
| Shyamalan, Gunaratnam; Ghosh, Koushik; Robinson, C. Michael; Jenkins, Paul J. Primary Arthroscopic Stabilization for a First-Time Anterior Dislocation of the Shoulder. Journal of Bone & Joint Surgery, American Volume Nov2008, Vol. 90-A Issue 11, p2550. | Arthroscopic |
| Simank, H.-G.; Dauer, G.; Schneider, S.; Loew, M. Incidence of rotator cuff tears in shoulder dislocations and results of therapy in older patients. Archives of Orthopaedic & Trauma Surgery May2006, Vol. 126 Issue 4, p235. | Rotator Cuff |
| Sivan, Manoj; Venkateswaran, Balachandran; Mullett, Hannan; Even, Tirtza; Khan, Saleem; Copeland, Steven; Levy, Ofer. Peripheral paresthesia in patients with subacromial impingement syndrome. Archives of Orthopaedic & Trauma Surgery Oct2007, Vol. 127 Issue 7, p609. | Rotator Cuff |
| Soliman, Omar A.; Koptan, Wael M.T. Proximal humeral fractures treated with hemiarthroplasty: Does tenodesis of the long head of the biceps improve results? Injury Apr2013, Vol. 44 Issue 4, p461. | Duplicate |
| Song, Joo-Hyoun; Lee, Joo-Yup; Chung, Yang-Guk; Park, Il-Jung. Distal interphalangeal joint arthrodesis with a headless compression screw: morphometric and functional analyses.  Archives of Orthopaedic & Trauma Surgery May2012, Vol. 132 Issue 5, p663. | Arthroscopic |
| Stapleton, Claire; Herrington, Lee; George, Keith. Sonographic evaluation of the subclavian artery during thoracic outlet syndrome shoulder manoeuvres. Manual Therapy Feb2009, Vol. 14 Issue 1, p19. | Radiology |
| Stein, T.; Buckup, J.; Mehling, A.; Hoffmann, R.; Efe, T.; Eisenhart-Rothe, R.; Welsch, F. Restoration of joint congruency and the glenoidal labrum after arthroscopic revision Bankart repair: a MRI match-paired analysis comparing primary Bankart repair and the uninjured labrum. Archives of Orthopaedic & Trauma Surgery Aug2014, Vol. 134 Issue 8, p1121. | Arthroscopic |
| Stein, T.; Buckup, J.; Welsch, F.; Efe, T.; Eisenhart-Rothe, R.; Hoffmann, R.; Zimmermann, E. Structural and clinical integrity of the rotator cuff in athletes after arthroscopic Bankart repair using the three-portal technique. Archives of Orthopaedic & Trauma Surgery Mar2015, Vol. 135 Issue 3, p369. | Arthroscopic |
| Stilling, Maiken; Mechlenburg, Inger; Amstrup, Anders; Soballe, Kjeld; Klebe, Thomas. Precision of novel radiological methods in relation to resurfacing humeral head implants: assessment by radiostereometric analysis, DXA, and geometrical analysis. Archives of Orthopaedic & Trauma Surgery Nov2012, Vol. 132 Issue 11, p1521. | Radiology |
| Rabuck, Stephen J.; Lynch, Jamie L.; Guo, Xin; Zhang, Li-Qun; Edwards, Sara L.; Nuber, Gordon W.; Saltzman, Matthew D. Biomechanical Comparison of 3 Methods to Repair Pectoralis Major Ruptures. American Journal of Sports Medicine Jul2012, Vol. 40 Issue 7, p1635. | Pectoralis Major |
| Strauss, Eric J.; Alaia, Michael; Egol, Kenneth A. Management of distal humeral fractures in the elderly. Injury Mar2007 Supplement 1, Vol. 38 Issue 3, p10. | Upper Extremity, Not Shoulder |
| Su, Brain W.; Solomons, Michael; Barrow, Andrew; Senoge, Matshediso E.; Gilberti, Marco; Lubbers, Lawrence; Diao, Edward; Quitkin, Matthew; Grafe, Michael W.; Rosenwasser, Melvin P. A Device for Zone-II Flexor Tendon Repair. Journal of Bone & Joint Surgery, American Volume Mar2006 Supplement 1, Vol. 88-A, p37. | Arthroscopic |
| Sung-Jae Kim; Sung-Hwan Kim; Su-Keon Lee; Jae-Wan Seo; Yong-Min Chun. Arthroscopic Repair of Massive Contracted Rotator Cuff Tears: Aggressive Release with Anterior and Posterior Interval Slides Do Not Improve Cuff Healing and Integrity. Journal of Bone & Joint Surgery, American Volume 8/21/2013, Vol. 95-A Issue 16, p1482. | Arthroscopic |
| Tashjian, Robert Z. Is there Evidence in Favor of Surgical Interventions for the Subacromial Impingement Syndrome? Clinical Journal of Sport Medicine Sep2013, Vol. 23 Issue 5, p406. | Rotator Cuff |
| Taverna E, Battistella F, Sansone V, Perfetti C, Tasto JP. Radiofrequency-based plasma microtenotomy compared with arthroscopic subacromial decompression yields equivalent outcomes for rotator cuff tendinosis. Arthroscopy. 2007 Oct;23(10):1042-51. PubMed PMID: 17916468. | Arthroscopic |
| Taylor, Drew W.; Petrera, Massimo; Hendry, Mike; Theodoropoulos, John S. A Systematic Review of the Use of Platelet-Rich Plasma in Sports Medicine as a New Treatment for Tendon and Ligament Injuries. Clinical Journal of Sport Medicine Jul2011, Vol. 21 Issue 4, p344. | Systematic Review |
| Theopold, Jan; Weihs, Kevin; Löffler, Sabine; Marquass, Bastian; Dercks, Nikolaus; Josten, Christoph; Hepp, Pierre. Image-free navigated coracoclavicular drilling for the repair of acromioclavicular joint dislocation: a cadaver study.  Archives of Orthopaedic & Trauma Surgery Aug2015, Vol. 135 Issue 8, p1077. | Cadaver |
| Thomas, Kristen; Litsky, Alan; Jones, Grant; Bishop, Julie Y. Biomechanical Comparison of Coracoclavicular Reconstructive Techniques. American Journal of Sports Medicine 04/01/2011, Vol. 39 Issue 4, p804. | Shoulder, Not Arthroplasty |
| Throckmorton TW, Gulotta LV, Bonnarens FO, Wright SA, Hartzell JL, Rozzi WB, Hurst JM, Frostick SP, Sperling JW. Pateint-specific targeting guides compared with traditional instrumentation for glenoid component placement in shoulder arthroplasty: a multi-surgeon study in 70 arthritic cadaver specimens. 2015. Journal of Shoulder and Elbow Surgery. | Cadaver |
| Todd, Jacquelyne; Scally, Andy; Dodwell, David; Horgan, Kieran; Topping, Annie. A randomised controlled trial of two programmes of shoulder exercise following axillary node dissection for invasive breast cancer. Physiotherapy Dec2008, Vol. 94 Issue 4, p265. | Cancer |
| van den Dolder, Paul Andrew; Ferreira, Paulo H.; Refshauge, Kathryn M. Effectiveness of soft tissue massage and exercise for the treatment of non-specific shoulder pain: a systematic review with meta-analysis. British Journal of Sports Medicine Aug2014, Vol. 48 Issue 16, p1216. | Systematic Review |
| Van der Meijden, O.A.; Marijin, H.R.; Hulsmans, M. et al. Operative treatment of dislocated midshaft clavicular fractures: plate or intramedullary nail fixation. 2015. Journal of Bone and Joint Surgery. | Clavicle |
| Vavken, Patrick; Sadoghi, Patrick; von Keudell, Arvind; Rosso, Claudio; Valderrabano, Victor; Müller, Andreas M. Rates of Radiolucency and Loosening After Total Shoulder Arthroplasty with Pegged or Keeled Glenoid Components. Journal of Bone & Joint Surgery, American Volume 2/6/2013, Vol. 95-A Issue 3, p215. | Systematic Review |
| Vermeulen, G.M.; Brink, S.M.; Slijper, H. et al. Trapeziometacarpal Arthrodesis or trapeziectomy with ligament reconstruction in primary trapeziometacarpal osteoarthritis. 2014. Journal of Bone and Joint Surgery. | Osteoarthritis |
| Virtanen, Kaisa J.; Remes, Ville; Pajarinen, Jarkko; Savolainen, Vesa; Bjorkenheim, Jan-Magnus; Paavola, Mika. Sling compared with plate osteosynthesis for treatment of displaced midshaft clavicular fractures. 2012. Journal of Bone and Joint Surgery. | Clavicle |
| Wang, Vincent M.; FanChia Wang; McNickle, Allison G.; Friel, Nicole A.; Yanke, Adam B.; Chubinskaya, Susan; Romeo, Anthony A.; Verma, Nikhil N.; Cole, Brian J. Medial Versus Lateral Supraspinatus Tendon Properties. American Journal of Sports Medicine 12/01/2010, Vol. 38 Issue 12, p2456. | Rotator Cuff |
| Wang, Cheng; Dai, Guofeng; Wang, Shaojin; Liu, Qi; Liu, Wenguang. The function and muscle strength recovery of shoulder after humeral diaphysis fracture following plating and intramedullary nailing. Archives of Orthopaedic & Trauma Surgery Aug2013, Vol. 133 Issue 8, p1089. | Shoulder, Not Arthroplasty |
| Wang A, McCann P, Colliver J, Koh E, Ackland Tm Joss B et al. Do postoperative platelet- rich plasma injections accelerate early tendon healing and functional recovery after arthroscopic supraspinatus repair? A randomized controlled trial. 2015. American Journal of Sports Medicine. | Duplicate |
| Wang A, McCann P, Colliver J, Koh E, Ackland Tm Joss B et al. Do postoperative platelet- rich plasma injections accelerate early tendon healing and functional recovery after arthroscopic supraspinatus repair? A randomized controlled trial. 2015. American Journal of Sports Medicine. | Arthroscopic |
| Weber, S.C.; Kauffman, J.I.; Parise, C,; et al. Platelet-rich fibrin matrix in the management of arthroscopic repair of the rotator cuff: a prospective, randomized, double-blinded study. 2013. American Journal of Sports Medicine. | Arthroscopic |
| Wei, David H.; Raizman, Noah M.; Bottino, Clement J.; Jobin, Charles M.; Strauch, Robert J.; Rosenwasser, Melvin P. Unstable distal radial fractures treated with external fixation, a radial column plate, or a volar plate: a prospective randomized trial. 2009. Journal of Bone and Joint Surgery. | Radius |
| Wellmann, Mathias; Wiebringhaus, Philipp; Lodde, Ina; Waizy, Hazibullah; Becher, Christoph; Raschke, Michael J.; Petersen, Wolf. Biomechanical evaluation of a single-row versus double-row repair for complete subscapularis tears. Knee Surgery, Sports Traumatology, Arthroscopy Dec2009, Vol. 17 Issue 12, p1477. | Rotator Cuff |
| Wilcke, Maria K T; Abbaszadegan, Hassan; Adolphson, Per Y. Wrist function recovers more rapidly after volar locked plating than after external fixation but the outcomes are similar after 1 year. Acta Orthopaedica Feb2011, Vol. 82 Issue 1, p76. | Wrist/Hand |
| Wood, Vanessa J.C.; Sabick, Michelle B.; Pfeiffer, Ron P.; Kuhlman, Seth M.; Christensen, Jason H.; Curtin, Michael J. Glenohumeral Muscle Activation During Provocative Tests Designed to Diagnose Superior Labrum Anterior-Posterior Lesions. American Journal of Sports Medicine Dec2011, Vol. 39 Issue 12, p2670. | Arthroscopic |
| Xie, Xuetao; Xie, Xiaoxing; Qin, Hui; Shen, Longxiang; Zhang, Changqing. Comparison of internal and external fixation of distal radius fractures. Acta Orthopaedica Jun2013, Vol. 84 Issue 3, p286. | Radius |
| Xuhui Liu; Laron, Dominique; Natsuhara, Kyle; Manzano, Givenchy; Kim, Hubert T.; Feeley, Brian T. A Mouse Model of Massive Rotator Cuff Tears. Journal of Bone & Joint Surgery, American Volume 4/4/2012, Vol. 94-A Issue 7, p601. | Animal |
| Yamada, Minoru; Higuchi, Takahiro; Morioka, Shu. Effect of Modified Mental Rotation Training on Patients with Frozen Shoulder. Rigakuryoho Kagaku 2009, Vol. 24 Issue 3, p459. | Physical Therapy |
| Yeon Soo Lee; Mihata, Terihisa; Joo Han Oh. Anatomically reproducible assessment of volumetric bone mineral density -- Based on clinical computed tomography. Journal of Biomechanics 2013, Vol. 46 Issue 4, p767. | Radiology |
| Yun, Mi; Oh, Joo; Yoon, Jong; Park, Sang; Hwang, Jung; Kil, Ho. Subacromial patient-controlled analgesia with ropivacaine provides effective pain control after arthroscopic rotator cuff repair. Knee Surgery, Sports Traumatology, Arthroscopy Oct2012, Vol. 20 Issue 10, p1967. | Arthro |
| Zehir, Sinan; Çalbıyık, Murat; Zehir, Regayip; Şahin, Ercan. Comparison of novel intramedullary nailing with mini-invasive plating in surgical fixation of displaced midshaft clavicle fractures.  Archives of Orthopaedic & Trauma Surgery Mar2015, Vol. 135 Issue 3, p339. | Clavicle |
| Zhang JH, Di ZL, He ZY, Feng JX, Xu RM. [Comparison of humeral head replacement and internal fixation for the treatment of 3 parts and 4 parts fractures of proximal humerus in the elderly]. Zhongguo Gu Shang. 2010 Jun;23(6):435-9. Chinese. PubMed PMID: 20669576. | Article Not Found |
| Zhang, Q., Zhou, J.; Ge, H.; Cheng, B. Tenotomy or tendesis for long head biceps lesions in shoulders with reparable rotator cuff tears: a prospective randomised trial. 2015. Knee Surgery, Sports Traumatology, Arthroscopy. | Rotator Cuff |
| Yiming Zhu; Yi Lu; Jiewei Shen; Jin Zhang; Chunyan Jiang. Locking Intramedullary Nails and Locking Plates in the Treatment of Two-Part Proximal Humeral Surgical Neck Fractures. Journal of Bone & Joint Surgery, American Volume 1/19/2011, Vol. 93-A Issue 2, p159. | Shoulder, Not Arthroplasty |
| Zooker, Chad C.; Parks, Brent G.; White, Kacey L.; Hinton, Richard Y. TightRope Versus Fiber Mesh Tape Augmentation of Acromioclavicular Joint Reconstruction. American Journal of Sports Medicine Jun2010, Vol. 38 Issue 6, p1204. | AC joint |
